# Intermediate-Type Vancomycin Resistance (VISA) in Genetically-Distinct *Staphylococcus aureus* Isolates Is Linked to Specific, Reversible Metabolic Alterations

**DOI:** 10.1371/journal.pone.0097137

**Published:** 2014-05-09

**Authors:** Elizabeth L. Alexander, Susana Gardete, Haim Y. Bar, Martin T. Wells, Alexander Tomasz, Kyu Y. Rhee

**Affiliations:** 1 Division of Infectious Diseases, Department of Medicine, Weill Cornell Medical College, New York, New York, United States of America; 2 Laboratory of Microbiology, The Rockefeller University, New York, New York, United States of America; 3 Department of Statistics, University of Connecticut, Storrs, Connecticut, United States of America; 4 Department of Biological Statistics and Computational Biology and Department of Statistical Sciences, Cornell University, Ithaca, New York, United States of America; 5 Department of Microbiology and Immunology, Weill Cornell Medical College, New York, New York, United States of America; University of Liverpool, United Kingdom

## Abstract

Intermediate (VISA-type) vancomycin resistance in *Staphylococcus aureus* has been associated with a range of physiologic and genetic alterations. Previous work described the emergence of VISA-type resistance in two clonally-distinct series of isolates. In both series (the first belonging to MRSA clone ST8-USA300, and the second to ST5-USA100), resistance was conferred by a single mutation in *yvqF* (a negative regulator of the *vraSR* two-component system associated with vancomycin resistance). In the USA300 series, resistance was reversed by a secondary mutation in *vraSR*. In this study, we combined systems-level metabolomic profiling with statistical modeling techniques to discover specific, reversible metabolic alterations associated with the VISA phenotype.

## Introduction

*Staphylococcus aureus* is a major human pathogen, responsible for significant morbidity and mortality [1]. The threat of *S. aureus* is due in large part to the emergence of antibiotic resistance [1]. For over 30 years, the glycopeptide antibiotic, vancomycin, has been the mainstay of therapy for treating methicillin-resistant *S. aureus* (MRSA) infections. Since 1997, however, an increasing number of MRSA strains have been reported to have decreased vancomycin susceptibility. Clinically, these vancomycin intermediate (VISA) strains are associated with an increased likelihood of treatment failure and complications [2].

From a microbiologic perspective, VISA-type vancomycin resistance is associated with a wide array of changes in bacterial physiology. These include an increase in cell wall diameter, altered autolysis, decreased growth, and alterations in gene transcription. Though the etiology of intermediate-level vancomycin resistance appears to be polygenetic (involving mutations in candidate loci *vraSR, yvqF, graSR*, and *walKR*, among others), resistant isolates from different genetic backgrounds share these pleiomorphic phenotypes [2]–[5]. Previous studies have described the physiologic, transcriptional and genetic changes associated with VISA-type resistance. However, little is known about what, if any, changes in bacterial metabolism might accompany VISA-type resistance. Such changes are of interest as alterations in nutrient availability, metabolism and environmental conditions have been shown to affect both the structure and composition of cell wall peptidoglycan [6], [7]. The aim of this study was to determine if VISA-type resistance in *S. aureus* might also be accompanied by specific changes in bacterial intermediary metabolism. To do so, we applied a conceptually unbiased, systems-level approach that combined metabolomic profiling with statistical modeling techniques.

Recent work reported a detailed genetic and physiologic analysis of VISA-type resistance in an isogenic series of methicillin-resistant *S. aureus* (MRSA) isolates [4]. The resistant strain – SG-R –the corresponding isogenic susceptible “parent” - SG-S – and its spontaneous revertant – SG-rev – all belonged to MRSA lineage ST8, pulsed-field gel electrophoresis (PFGE) type USA300. Moreover, the resistant isolate was found to differ from the parent and revertant strains with respect to its *in vitro* rate of growth, cell wall diameter, autolytic properties and antibiotic resistance profile; all of which were reverted – i.e., returned to the phenotype of the parental strain – in the spontaneous revertant. Whole genome sequencing of these 3 strains, followed by complementation analysis, identified an amino acid change A165D (alanine to aspartate) in the genetic determinant *yvqF* (a negative regulator of the two-component regulator *vraSR*) as the genetic lesion responsible for vancomycin resistance in SG-R. These complementation experiments further showed that this specific mutation was responsible for the physiologic abnormalities detected in strain SG-R, described above. Additional complementation experiments further established the subsequent mutation in *vraSR* (the target of *vyqF* inhibition) was responsible for the loss of resistance in the revertant strain, SG-rev [4]. These three isolates thus provided a unique, genetically-defined window into VISA-type resistance. In particular, the presence of the revertant isolate afforded the potential opportunity to discover metabolic alterations specifically linked to the *yvqF* allele.

The JH series consists of 5 sequential, clonal MRSA isolates (JH1, JH2, JH5, JH6 and JH9) recovered from a single patient in which VISA-type resistance emerged in the setting of extensive vancomycin chemotherapy. Whole genome sequencing revealed the appearance of 36 point mutations associated with an increase in vancomycin MIC from JH1 (MIC 1.0 µg/mL) to JH9 (MIC 8 µg/mL) [8]. To determine the extent to which the metabolic changes observed in SG-R versus SG-S were specific to the *yvqF*-associated VISA phenotype, rather than genetic lineage, we compared their metabolic profiles to the VSSA isolate, JH1, and a representative VISA isolate from the series, JH2. JH2 was chosen as the level of vancomycin resistance (as defined by MIC) is closest to that of SG-R (4 µg/mL and 3 µg/mL, respectively). Recent complementation experiments (Gardete, *et al.*, in preparation) indicate that the mutation responsible for vancomycin resistance in JH2 is an amino acid change H164R (histidine to arginine) in *yvqF*, only one amino acid away from the resistance-conferring mutation in SG-R [8].

Given the multiple phenotypic changes common to VISA isolates of different genetic lineages, we hypothesized that VISA-type resistance might also be accompanied by specific changes in *S. aureus* intermediary metabolism. We therefore examined the baseline metabolic profiles of isolates SG-S, SG-R and SG-rev, as well as JH1 and JH2 during the logarithmic phase of growth using a mass spectrometric metabolomics platform to determine what, if any, metabolic changes are common to the *yvqF*-associated VISA phenotype.

## Methods

### Sample Preparation and Liquid Chromatography-Mass Spectrometry (LC/MS)

Sample preparation and liquid chromatography-mass spectrometry analyses were performed using methods similar to those previously detailed by de Carvalho *et al* [9]. Briefly, single colony units of each isolate from 24 h growth on brain heart agar (BHA, Difco Laboratories, Detroit MI) at 37°C were selected and again grown on BHA at 37°C for 24 h growth to obtain a clonal population and synchronize the growth cycle. A 0.1 OD_595_nm suspension of each was then made in Brain Heart Infusion (BHI) (Difco Laboratories, Detroit MI), from which 250 µL was inoculated to 5 mL fresh BHI to obtain a .005 OD starting culture. Liquid cultures were grown to early stationary phase (8 h) at 37°C with aeration (220 rpm) and then inoculated (500 µL) onto 22 mm nitrocellulose filters (Millipore, Billerica, MA) under vacuum filtration in sterile conditions, according to Brauer *et al* [10]. Biological replicates (at least 4) of *S. aureus*-laden filters were then placed atop equivalent agar media (BHA) and allowed to grow at 37°C. Bacterial growth was monitored by weighing additional biological replicates of *S. aureus* laden filters (which were then sacrificed at 24 hours) which increased with constant doubling times from 1 h to 12 h, and a consistent mid-log-rhythmic phase of 4–5 h. Filters were harvested at mid-log-rhythmic phase (4 h) and metabolically quenched by immersion into acetonitrile/methanol/H20 (40:40:20) precooled to −40°C. Metabolites were extracted by mechanical lysis of the entire solution with 0.1 mm Zirconia beads in a Precellys 24 tissue homogenizer (Bertin Technologies, France) for 4 cycles of 30 s at 6000 rpm with 2 minute cooling intervals at 0°C. Lysates were clarified by centrifugation at 14 g for 10 minutes at 4°C and extracted 50:50 into acetonitrile with 0.2% formic acid. Intracellular metabolites were analyzed by liquid chromatography-mass spectrometry as recently described by Eoh and Rhee [11]. Experiments were performed at least in duplicate to ensure replicability.

### LC/MS Data Processing and Analysis

Metabolites were searched for both by chemical formula and molecular feature and identities of specific metabolites were confirmed against pure chemical standards (where available) by molecular mass (mass tolerance <0.01Da) and retention times. Where chemical standards were not available, provisional identifications were made by matching against a database of accurate mass-retention time pairs using Agilent MassHunter Qualitative Analysis software [12]. Peak heights of all detected metabolites were imported into an Excel (Microsoft) data sheet, and adjusted for bacterial biomass by residual protein content analysis (Pierce BCA Protein Assay Kit, Thermo Scientific, Rockford, IL), as described by Stich *et al* [13]. Normalized levels for each metabolite across replicates from independent experiments were generated by dividing the adjusted abundance for each by the average adjusted abundance in the parent VSSA to allow comparison across metabolites (Tables S1–S4). A heat map was then created using Microsoft Excel after log(2) transformation of the average normalized abundance of each metabolite in each isolate (average of replicates across independent experiments).

### Data Normalization and Processing

To minimize batch effects associated with individual experiments, we normalized the data from each experimental repeat to the first experiment. To do so, we calculated the average of the data for every isolate (SG-S, SG-R, SG-rev, JH1, JH2) in each experiment, and divided this by the average of the data for the same isolate obtained on the first experimental repeat. We then multiplied the values obtained on each subsequent experimental repeat by this ratio to obtain values normalized to the first experiment.

### Hierarchical Mixture Model Analyses

Changes in bacterial physiology may lead to the adoption of different homeostatic states. We therefore hypothesized that VISA isolates might exhibit changes in the mean abundance of individual metabolites. On the other hand, we considered that changes in bacterial physiology might also activate new regulatory mechanisms that seek to maintain metabolite pool sizes and manifest only as changes in flux. We therefore sought to detect changes in both metabolite abundance and flux (as reported by pool size variations).

Conventional t-test is one of the most commonly used methods to compare metabolite levels between two groups. This method is only valid, however, if certain assumptions hold. First, the distribution of data must be approximately normal, or the sample size must be sufficiently large. Second, the difference between the means of the two groups (D) and the standard error of that difference (S) must be independent. Finally, the variances must be equal in the two groups. In our analyses, our normalized data violated these assumptions. While the means were normalized with the first set as reference, within each group the means were quite different, and there appeared to be batch effects, as seen in Figures S1 and S2. Additionally, there appeared to be a relationship between means and variances, and variances in the two groups were sometimes quite different. To address the issues of different means, batch effects and the relationship between mean and variance, we median centered the data (Figure S1 & S2). To address the problem of unequal variance, one option would be use of a modified t-test with a Satterthwaite approximation to the transformed data. However, as we desired to simultaneously test for changes in variance, as well as adjust for them (as these could be due to changes in flux), we chose to use a more powerful approach. We therefore used a newly-developed hierarchical mixture model, which benefits from “borrowing information” across tests [14]. The phrase “borrowing information” refers to methods that combine data from multiple tests (metabolites) into a simple parsimonious model, effectively increasing the sample size. Specifically, in the model used here, we assumed that the differences between means, D_i_ (i  =  1,2,3,…72), are observations from up to three normal components – non-differential, increased abundance, or decreased abundance. Thus, metabolites in each component are all used jointly to estimate the difference between population means in that component. Moreover, this approach allows the two groups to have different variances. The 72 ratios of the variances of the two groups are also assumed to be a mixture of up to three components (same variance in both groups, larger variance in group 1, or larger variance in group 2). This leads to further improvement in power, as a result of better estimation of the standard errors S_i_ (the denominators of the t-statistics.)

We refer to this approach, in which we test whether the two groups have different variances, different means, or both, as the “combined analysis of mean abundance and variance” [14]. We refer to the analysis of means alone (assuming homogeneity of variance) as “analysis of changes in mean abundance” [14]. Simulation studies show that this hierarchical mixture model provides significant improvement over methods that either test one metabolite at a time, controlling for Type-I error probability or false discovery rate (such as the simple T-test or Satterthwaite's approximation).

### Statistical Analyses

Biomass-adjusted, baseline-normalized and experiment-normalized ion counts (provided in Tables S3 & S4) were recorded in a Microsoft Excel database and plotted in R [15]. Results of these plots showed that, despite the means being normalized between experiments, within each group the means were different and batch effects persisted. To adjust for this, the data was centered to equalize the means across groups, as previously described (Figure S1 & S2). Figure S3 demonstrates similar output statistics (minimum, quartiles, mean, variance and coefficient of variation) between the groups tested (JH2 versus JH1, SG-R versus SG-S, etc.) using the mean-centered, normalized data. Resultant overall differences between means and variances between the susceptible and resistant isolates for each series were incorporated as the hypothesized mean and variance and differences in the mean abundance of each metabolite were tested for using the hierarchical mixture model described above [14], and adjusted for multiple hypothesis testing by the Benjamini-Hochberg procedure [16]. Combined analysis of changes in mean abundance and variance was then performed as previously described, again correcting for multiple hypothesis testing by the Benjamini-Hochberg procedure [16]. In addition, analyses of changes in mean abundance was performed by Significance Analysis of Microarrays for Excel (SAM) analysis (with input parameters set to two class unpaired, FDR < 1%, median centered) as previously described [17]. Hierarchical cluster analysis of the 72 identified metabolites across all samples for the JH series and SG series was performed using TM4MEV, a freeware software program for multi-parametric statistical analysis using a Pearson correlation distance metric (after standardizing by metabolite and group) [18]. Finally, principal component analysis was performed in R (using the default function princomp) on the 72 identified metabolites across all samples for each series individually and together to determine the extent to which metabolic changes clustered according to strain background, resistance phenotype, or did not cluster [15]. Unless otherwise cited, metabolites were ascribed to pathways by comparison against the KEGG (Kyoto Encyclopedia of Genes and Genomes) Database [19].

## Results

### Analysis of the JH Series Yields a Greater Number of Metabolic Alterations than Analysis of the SG Series, Consistent with a Greater Number of Genetic Mutations

We detected and quantified a maximum of 228 metabolites (defined as a chromatographically resolved family of co-eluting ions corresponding to an empirically confirmed molecular formula), of which 164 were observed in all three SG isolates (SG-S, SG-R and SG-rev) in three independent experiments (Table S1). A schematic representation of the metabolic profile (LC-MS overlay) of each isolate is shown in Figure S4. Of the 164 “core” metabolites detected in all three SG isolates, we were able to provisionally assign and/or chemically confirm the identities of 72, using an accurate mass-retention time database [12]. These 72 identified metabolites included intermediates of glycolysis/gluconeogenesis/the pentose phosphate pathway, fermentation, the tri-carboxylic (TCA) acid cycle, amino acid metabolism, pyrimidine metabolism, purine metabolism, and others [20]. While the identities of the remaining 92 metabolites were not definitively confirmed, 19 exhibited statistically significant differences in their intracellular abundance between SG-R and SG-S by hierarchical mixture model analysis [14]. Of these 19, 4 returned towards the levels of the parental strain (albeit imperfectly in some cases) in SG-rev, indicating a possible link to the VISA phenotype (Table S1).

Metabolomic profiling of JH2 and its parental isolate JH1 again yielded the same 72 metabolites as the SG series whose identity could be confirmed using accurate mass/retention time identifiers (Table S2). Comparison of the intracellular abundance of these metabolites in the VISA (JH2) against the parent VSSA (JH1), however, revealed a larger number of alterations than found in SG-R compared to its parental isolate, SG-S by both statistical methods (SAM and hierarchical mixture modeling). These results are consistent with the larger number of genetic mutations in the JH isolates (8 in JH2 vs. JH1 and 5 in SG-R vs. SG-S). Specifically, hierarchical mixture model analysis of the SG series yielded 12/72 metabolites whose mean abundance was significantly different (≥0.25-fold) between SG-R and SG-S (*p*≤0.05), after adjusting for multiple comparisons using the Benjamini-Hochberg correction (Table S1). Of these 12, eleven metabolites were significantly altered on analysis of both mean abundance and variance, after adjusting for multiple comparisons. Hierarchical mixture model analysis of the JH series, by contrast, identified 25/72 metabolites whose mean abundance was significantly different between JH2 and JH1 (≥0.25-fold, *p*≤0.05) after adjusting for multiple comparisons. Of these 25 metabolites, 22 metabolites remained significantly different on combined analysis of mean abundance and variance (again adjusted for multiple comparisons) and 13 were unique to the JH series (i.e. remained unaltered in the SG series) (Tables S1 & S2). Analysis by SAM similarly yielded a larger number of altered metabolites in the JH series than the SG-series, consistent with the larger number of genetic alterations. Specifically, 50/72 identified metabolites showed changes in intracellular abundance by SAM analysis in the JH series versus 26/72 in the SG series (Tables S1 & S2).

### Principal Components and Hierarchical Cluster Analyses Show Segregation of Isolates by Resistance Phenotype

**Figure 1:**
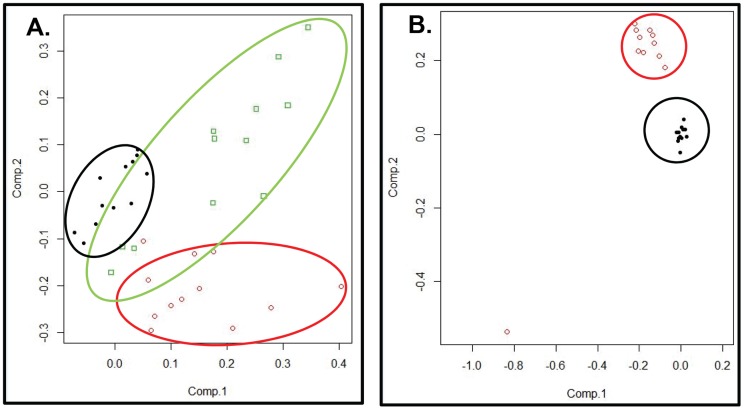
Principal component analyses of the metabolic profiles of the SG series (A) and the JH series (B) show clustering of replicates by resistance phenotype. (**A**) Principal component analysis of the metabolic profiles of isolates from the SG series (SG-S, SG-R and SG-rev) shows a separation of samples (replicates) by resistance phenotype. Specifically, the metabolic profiles corresponding to replicates of the VISA strain, SG-R (red ellipse) cluster separately from those of its parent VSSA, SG-S (black ellipse), while those of SG-rev (green ellipse) cluster between these two, overlapping both. (**B**) Principal component analysis of the metabolic profiles of isolates from the JH series again shows clustering of replicates by phenotype, with both the parent VSSA JH1 (black circles) and VISA JH2 (red circles) forming non-overlapping clusters (though one outlier, JH2-10, is noted).

**Figure 2:**
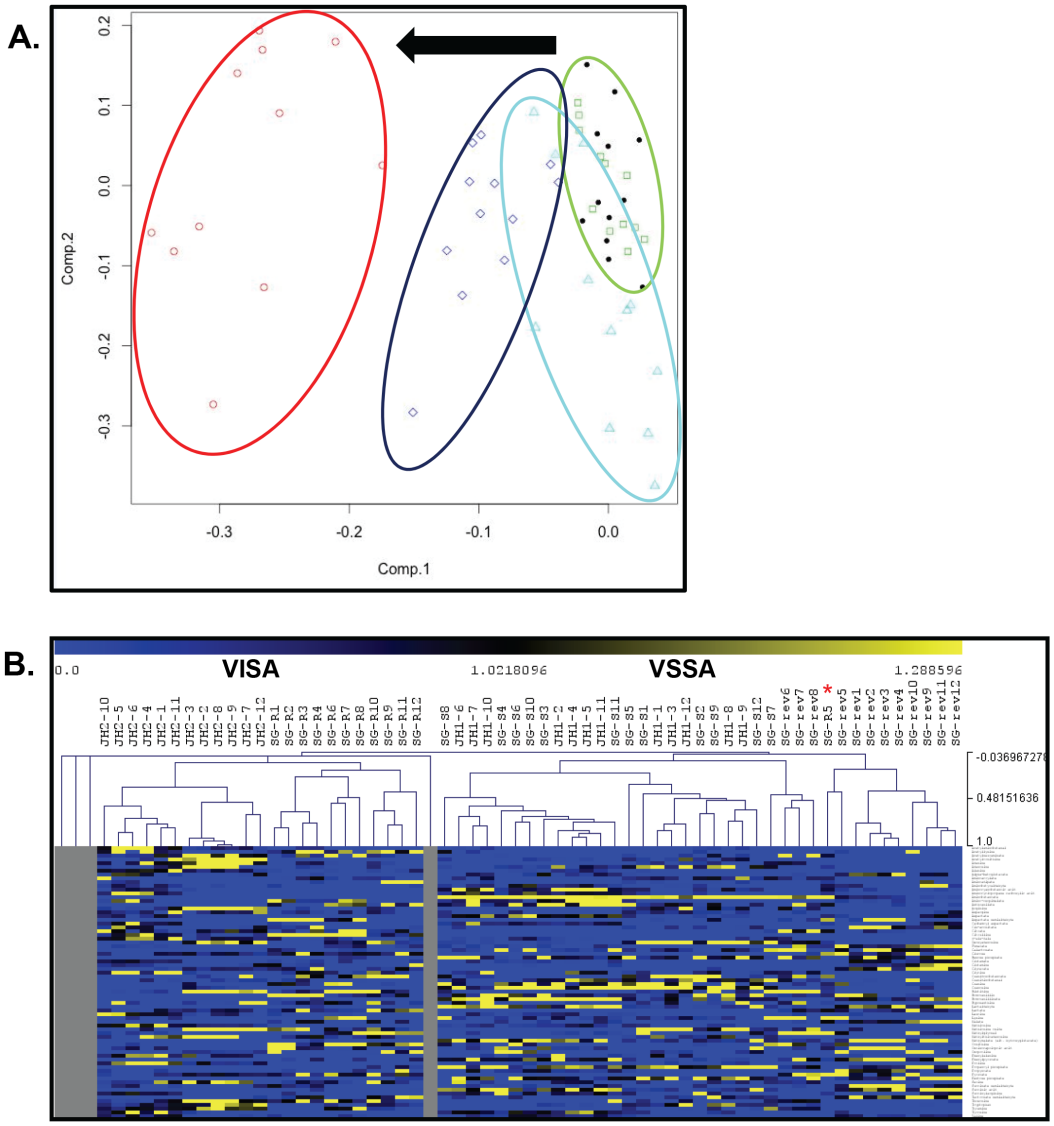
Principal component and hierarchical cluster analyses show separation of isolates by resistance phenotype. Principal component analysis **(A)** of the metabolic profiles of all isolates from both series again shows a separation of isolates by resistance phenotype. Specifically, the metabolic profiles of parent VSSA isolates SG-S (green squares) and JH1 (black dots) form a single cluster (green ellipse), while those corresponding to VISA isolates SG-R (dark blue diamonds) and JH2 (red circles) form two separate clusters (dark blue ellipse and red ellipse, respectively) along the same vector of change (denoted by the black arrow) to the left of the VSSA cluster. The metabolic profiles corresponding to the revertant SG-rev (light blue diamonds) cluster between the susceptible parent (SG-S) and resistant (SG-R) isolates, consistent with its revertant phenotype (light blue ellipse). **(B)** Results of hierarchical cluster analysis also show that the metabolic profiles of all five isolates from both series separate by resistance phenotype, forming two distinct branches with the VISA isolates JH2 and SG-R on the left and VSSAs SG-S, JH1 and SG-rev on the right, though one replicate of SG-R (SG-R5) was found to cluster with the parental VSSA isolates (red asterix).

Comparison of the metabolic profiles of isolates SG-S, SG-R and SG-rev by principal component analysis (PCA) of the 72 identified metabolites demonstrated clustering of samples by resistance phenotype (Figure 1A). Specifically, the metabolic profiles of SG-S and SG-R formed separate clusters on PCA, while those of SG-rev clustered between these two groups (Figure 1A), suggesting that some, but not all of the metabolic changes associated with acquisition of vancomycin resistance in SG-R reversed with the loss of resistance in SG-rev. Principal component analysis of the metabolic profiles of the JH isolates showed a similar segregation of samples by resistance phenotype with JH1 and JH2 forming distinct clusters, though one outlier (JH2-sample 10) was noted (Figure 1B). Notably, on principal component analysis of all isolates from both series, we again noted clustering according to resistance phenotype. Specifically, the metabolic profiles of the “parent” vancomycin susceptible isolates, JH1 and SG-S, formed a single cluster (Figure 2A, green ellipse) while those of SG-S and JH-2 formed separate clusters (red and dark blue ellipses, respectively) along the same vector of change (as denoted by the black arrow) from the cluster corresponding to the susceptible isolates. As seen in the principal component analysis of the SG series alone, the metabolic profiles of the SG-rev isolates again clustered between those of its susceptible parent (SG-S) and resistant (SG-R) isolates, overlapping both.

We also noted a separation of the metabolic profiles of all five isolates from both series according to resistance phenotype on hierarchical cluster analysis (HCA) [18]. Specifically, on HCA of the 72 identified metabolites in all isolates, SG-R and JH2 separated into one branch, while SG-S, JH1 and SG-rev separated into another (Figure 2B), though one replicate of SG-R (SG-R5) did cluster with the susceptible isolates (red asterix). As seen on principal component analysis, SG-S and JH1 samples were somewhat mixed, while the SG-rev samples clustered together, though still within the same branch as the parent VSSA isolates, consistent with its revertant phenotype.

### Vancomycin Resistance is Associated with Specific Metabolic Alterations, Common to Both SG-R and JH2

**Figure 3:**
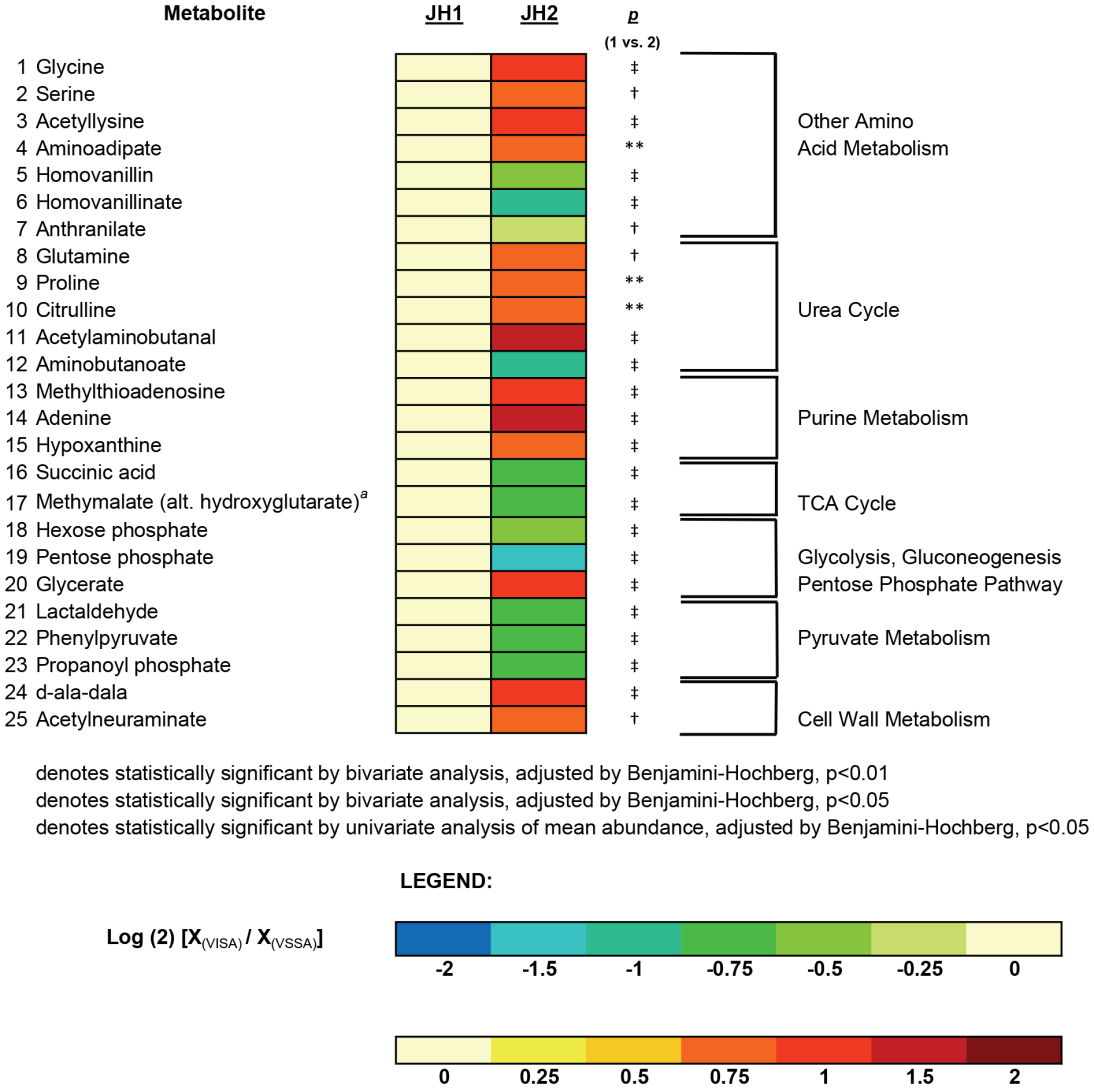
Heat map of Altered Metabolites in the VISA, JH2, Versus its Parent VSSA, JH1. Heat map displaying the 25 metabolites whose abundance was significantly altered in the VISA isolate, JH2, compared against its parent VSSA, JH1. Changes in abundance are indicated by color coding with red indicative of increases in mean intracellular abundance relative to the baseline (defined by the abundance in JH1) and blue indicative of decreases in intracellular abundance on a log (2) scale. Specific *p*-values for the comparison of JH2 versus JH1 are denoted to the near right of the heat map. Metabolites are grouped according to pathway, denoted to the far right of each metabolite. The superscript (*a*) denotes unable to determine if methylmalate or hydroxyglutarate in the absence of a chemical standard.

**Figure 4:**
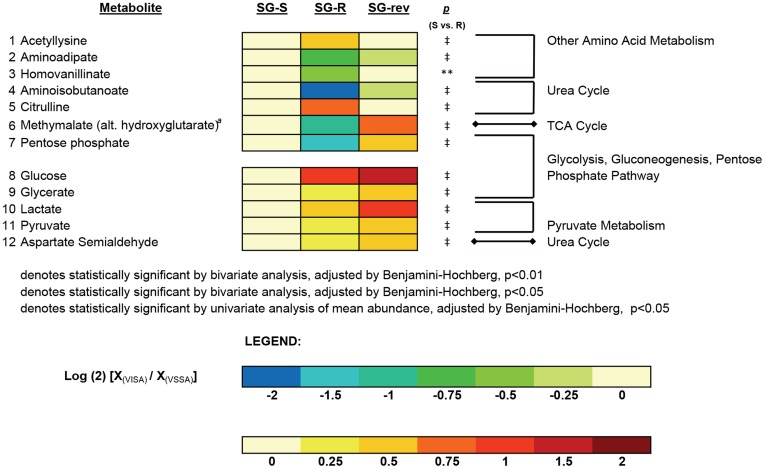
Heat Map of Altered Metabolites in the VISA, SG-R, Versus its Parent VSSA, SG-R and the revertant VSSA, SG-rev. Heat map displaying the 12 metabolites whose abundance was significantly altered in the VISA isolate, SG-R compared against the parent VSSA, SG-S. Changes in abundance are indicated by color coding with red indicative of increases in mean intracellular abundance relative to the baseline (defined by the abundance in SG-S) and blue indicative of decreases in intracellular abundance on a log (2) scale. Specific *p*-values for the comparison of SG-R versus SG-S are denoted to the near right of the heat map. Metabolites are grouped according to pathway, denoted to the far right of each metabolite. Metabolites 1–7 reversed directionality in the revertant isolate (SG-rev) indicating a link to vancomycin resistance. Metabolites 8–12, however, did not reverse in SG-rev and are separated by a row to denote this difference. The superscript (*a*) denotes unable to determine if methylmalate or hydroxyglutarate in the absence of a chemical standard.

Specific metabolites whose abundances were altered in JH2 compared against JH1 on hierarchical mixture modeling included intermediates of the urea cycle, purine metabolism, the tri-carboxylic acid (TCA) cycle, glycolysis/gluconeogenesis/pentose phosphate pathway, pyruvate metabolism, and cell wall metabolism, among others (Figure 3). The 12 metabolites whose abundance was significantly altered in the SG series by hierarchical mixture modeling also included intermediates of the urea cycle, TCA cycle, glycolysis/gluconeogenesis/pentose phosphate pathway and pyruvate metabolism, of which seven subsequently reversed in the direction of the parental isolate (albeit sometimes imperfectly) in the revertant, SG-rev (Figure 4).

**Figure 5:**
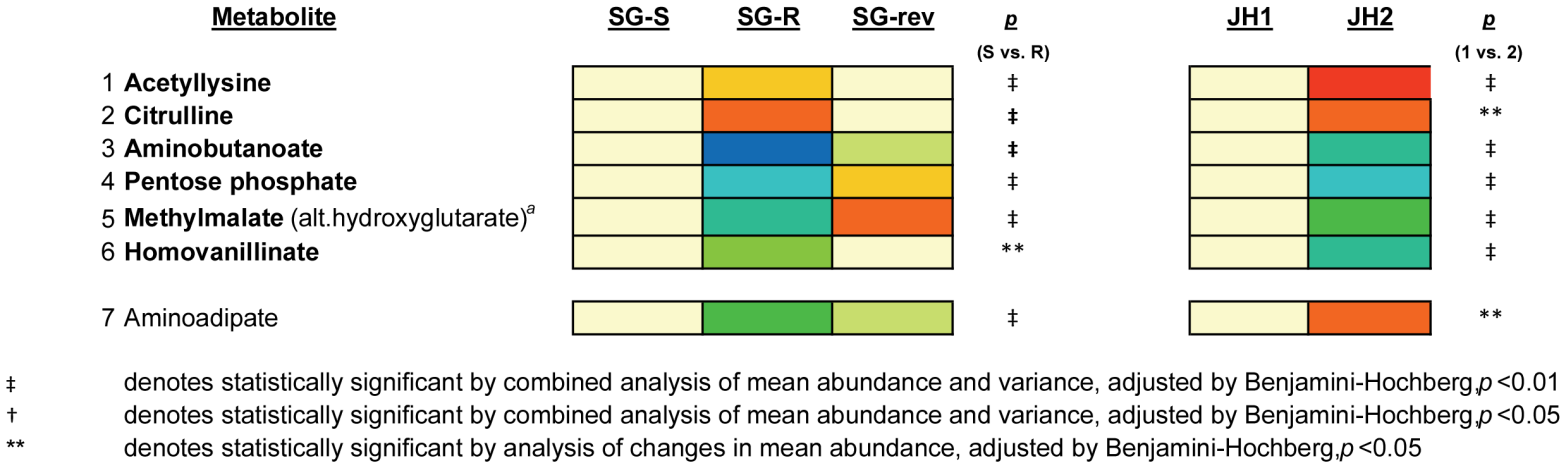
Heat Map of Metabolites In Which Alterations in Intracellular Abundance was Linked to VISA-type Resistance. Heat map displaying the 7 metabolites whose abundance was significantly altered in the VISA isolates from both series (SG-R and JH2) compared against the parent VSSA isolates (SG-S and JH1) and reversed directionality in the revertant, SG-rev, indicating a link to the vancomycin resistance phenotype. Of these seven metabolites, all but one (aminoadipate) changed in a similar fashion in both VISA isolates compared against the parent VSSA isolates. Changes in abundance are indicated by color coding with red indicative of increases in mean intracellular abundance relative to the baseline (defined by the abundance in SG-S) and blue indicative of decreases in intracellular abundance on a log (2) scale. Specific *p*-values for the comparison of SG-R versus SG-S are denoted to the near right of the heat map. The superscript (*a*) denotes unable to determine if methylmalate or hydroxyglutarate in the absence of a chemical standard.

Of the 7 metabolites whose intracellular abundance was significantly altered in both series of isolates on hierarchical mixture modeling, and returned in the directionality of baseline in the revertant, SG-rev (albeit sometimes imperfectly), 6 changed in the same direction in the VISA isolates from both series (SG-R or JH2) when compared against their respective parental VSSA strain (i.e. metabolites 1–2 increased in both VISAs and metabolites 3–6 decreased in both VISAs) (Figure 5). Results of SAM analyses similarly yielded 11 metabolites whose abundance was significantly altered in both VISA isolates and returned in the direction of baseline in the revertant, SG-rev. Of these 11, ten were altered in the same direction in the VISA isolate from both series (Figure S5). All six metabolites which were similarly altered in both VISAs on hierarchical mixture model analyses were also altered on SAM analyses, and included intermediates involved in three metabolic pathways – the urea cycle (aminobutanoate, citrulline) pentose phosphate pathway (pentose phosphate) and the TCA cycle (methylmalate) - among others [20], [21] (Figure 5, Figure S5). As the abundance of these 6 metabolites was significantly altered in a similar manner in the VISA isolate in both series and returned towards levels of the parental isolate in the revertant, they most likely represent the metabolic correlates of *yvqF*-associated VISA-type resistance.

## Discussion

This study extends what is known about the VISA phenotype in *S. aureus* to include specific and reversible alterations in *S. aureus* intermediary metabolism. Pathways of intermediary metabolism provide the biosynthetic precursors, ATP, and reducing equivalents used by all cellular processes. It is thus remarkable that natural VISA-type resistance emerged with such a limited, but specific, impact on the intermediary metabolism of *S. aureus.* It is also noteworthy that despite a limited number of metabolic alterations associated with the VISA phenotype, the metabolic profiles of these isolates segregated according to resistance on both principal component analysis and hierarchical cluster analyses.

Additional studies will be required to more fully characterize the complement of metabolic changes associated with VISA-type resistance, particularly in isolates with other resistance-conferring mutations (e.g. *graRS*, *walKR, rpoB* and *pp2C*), as well as their mechanistic significance [2]. However, it is interesting that despite differences in the number of altered metabolites, both isolate series showed alterations in intermediates of the urea cycle, TCA cycle and pentose phosphate pathway. It is also of interest to note that citrulline and aminobutanoate, the two metabolites whose intracellular abundances were most increased and decreased, respectively, are both intermediates of the urea cycle, which generates substrates that may feed pyrimidine synthesis, which has been linked to cell wall metabolism [22], [23]; and arginine metabolism, which has also been implicated in the unique ability of USA300 *S. aureus* strains to survive the acidic environment of the skin [24].

The current study thus provides unexpected evidence of a specific link between alterations in core metabolism and the VISA phenotype. The metabolic pathways involved, the specific ways in which they are altered, and whether/how those alterations may support vancomycin resistance remain to be explored. The discovery of other metabolic changes apparently dissociated from vancomycin resistance more broadly highlights a generally unrecognized class of physiologic changes associated with the acquisition and/or evolution of antibiotic resistance and/or adaptation to the host environment.

## Supporting Information

Figure S1 **Box-plot of the distribution of normalized data for the SG series.** A box-plot (A) of the distribution of the normalized data for the SG series show that, despite the means being normalized, within each group the means are different and batch effects persist. To adjust for unequal means and batch effects within groups we centered the data (B) so all samples have the same means.
(PDF)

Figure S2 **Box-plot of the distribution of normalized data for the JH series.** Box plot (A) of the distribution of the normalized data for the JH series similarly shows that despite normalization, within each group the means are different. Box plot (B) shows the distribution after centering the data.
(PDF)

Figure S3 **Summary statistics for all analyses performed using hierarchical mixture modeling.** **(A)** Displays the output summary statistics, including minimum, quartiles, maximum, mean, variance and coefficient of variance (CV) from the analysis of the 72 named metabolites in isolates JH1 (WT1-12) and JH2 (M1-12). Notice that the overall distributions of the metabolites are very similar across individuals and groups. **(B)** Show the same output summary statistics from analysis of the 72 named metabolites in isolates SG-S versus SG-R and SG-R versus SG-rev.
(DOCX)

Figure S4 **Metabolomic comparisons of isogenic VSSA (SGS, SG-rev, JH1) and VISA (SG-R, JH2) strains demonstrate unique and specific metabolic changes.** Schematic of data output from a representative LC-MS analysis of each isolate. Data correspond to individual metabolites represented by individual points on a three dimensional axis where the x-axis denotes chromatographic retention time in minutes (RT), z-axis denotes accurate mass in atomic mass units (mass) and y-axis denotes peak height in total ion counts, which can be used as an estimate of abundance (abundance). 186 unique metabolites were detected and quantified in **SG-S (blue dots)**, compared against 228 in **SG-R (red dots)** and 220 in **SG-rev (dark blue dots)**. A total of 164 metabolites were common to all three isolates in three independent experiments.
(TIF)

Figure S5 **Heat map displaying the 11 metabolites whose abundance was significantly altered on SAM analysis (> 0.25-fold, FDR < 1) in the VISA, SG-R compared against the parent VSSA SG-S, and reversed directionality in SG-rev.** Of these eleven metabolites, ten were significantly altered in a similar direction in the VISA isolate JH2 compared against its parent VSSA JH1. Changes abundance are indicated by color coding with red indicative of increases in mean intracellular abundance relative to the baseline (defined by the abundance in SG-S) and blue indicative of decreases in intracellular abundance on a log (2) scale. Bold font denotes the six metabolites whose abundance was also significantly altered in both VISA isolates on hierarchical mixture model analysis.
(TIF)

Table S1 **List and characteristics of the 164 “core” metabolites (including 72 identified) in isolates SG-S, SG-R and SG-rev.** Superscript (^a^) denotes Retention Time (RT), (^b^) denotes Positive Mode (POS) versus Negative Mode (NEG), (^c^) denotes Molecular Feature Extraction (MFE), which extracts chromatographic peaks by molecular features versus Find by Formula (FBF) which extracts peaks by chemical formula. Superscript (^d^) denotes metabolites confirmed by chemical standard (stnd). All other identifications are provisional identifications made by matching against a database of accurate mass-retention time pairs (mass-matching, MM), (^e^) denotes univariate statistical analysis of changes (> 0.25 – fold) in mean intracellular abundance by hierarchical modeling, adjusted by Benjamini-Hochberg procedure, (^f^) denotes bivariate analysis of changes (> 0.25 – fold) in intracellular abundance and variance by hierarchical modeling, adjusted by Benjamini-Hochberg procedure. Significance analysis of microarrays (SAM) analysis of changes in intracellular abundance (> 0.25-fold, FDR < 1%), significant metabolites denoted by an asterix (*). **Bold font** is used to indicate those metabolites whose abundance was altered in a similar fashion in the VISA isolate from both series (SG-R and JH2) and subsequently reversed in the revertant, SG-rev, as shown in Figure 4.
(PDF)

Table S2 **List and characteristics of the 72 identified metabolites for isolates JH1 and JH2.** Superscript (^a^) denotes Retention Time (RT), (^b^) denotes Positive Mode (POS) versus Negative Mode (NEG), (^c^) denotes Molecular Feature Extraction (MFE), which extracts chromatographic peaks by molecular features versus Find by Formula (FBF) which extracts peaks by chemical formula. Superscript (^d^) denotes metabolites confirmed by chemical standard (stnd). All other identifications are provisional identifications made by matching against a database of accurate mass-retention time pairs (mass-matching, MM), (^e^) denotes univariate statistical analysis of changes (> 0.25 – fold) in mean intracellular abundance by hierarchical modeling, adjusted by Benjamini-Hochberg procedure, (^f^) denotes bivariate analysis of changes (> 0.25 – fold) in intracellular abundance and variance by hierarchical modeling, adjusted by Benjamini-Hochberg procedure. Significance analysis of microarrays (SAM) analysis of changes in intracellular abundance (> 0.25-fold, FDR < 1%), significant metabolites denoted by an asterix (*). **Bold font** is used to indicate those metabolites whose abundance was altered in a similar fashion in the VISA isolate from both series (SG-R and JH2) and subsequently reversed in the revertant, SG-rev, as shown in Figure 4.
(PDF)

Table S3 **Characteristics and “raw” (biomass-adjusted and normalized to baseline) ion counts of the 164 “core” metabolites (including 72 identified) in isolates SG-S, SG-R and SG-rev.** Superscript (^a^) denotes Retention Time (RT), (^b^) denotes Positive Mode (POS) versus Negative Mode (NEG), (^c^) denotes Molecular Feature Extraction (MFE), which extracts chromatographic peaks by molecular features versus Find by Formula (FBF) which extracts peaks by chemical formula.
(XLSX)

Table S4 **Characteristics and “raw” (biomass-adjusted and normalized to baseline) ion counts of the 72 identified metabolites for isolates JH1 and JH2.** Superscript (^a^) denotes Retention Time (RT), (^b^) denotes Positive Mode (POS) versus Negative Mode (NEG), (^c^) denotes Molecular Feature Extraction (MFE), which extracts chromatographic peaks by molecular features versus Find by Formula (FBF) which extracts peaks by chemical formula.
(XLSX)
