# Supplementary material for: Intermediate-Type Vancomycin Resistance (VISA) in Genetically-Distinct Staphylococcus aureus Isolates Is Linked to Specific, Reversible Metabolic Alterations
Source: PLoS One. 2014 May 9;9(5):e97137. doi: 10.1371/journal.pone.0097137 (PMC4016254; doi:10.1371/journal.pone.0097137)
Supplement: Figure S1 — Box-plot of the distribution of normalized data for the SG series. A box-plot (A) of the distribution of the normalized data for the SG series show that, despite the means being normalized, within each group the means are different and batch effects persist. To adjust for unequal means and batch effects within groups we centered the data (B) so all samples have the same means. (PDF) [file pone.0097137.s001.pdf]

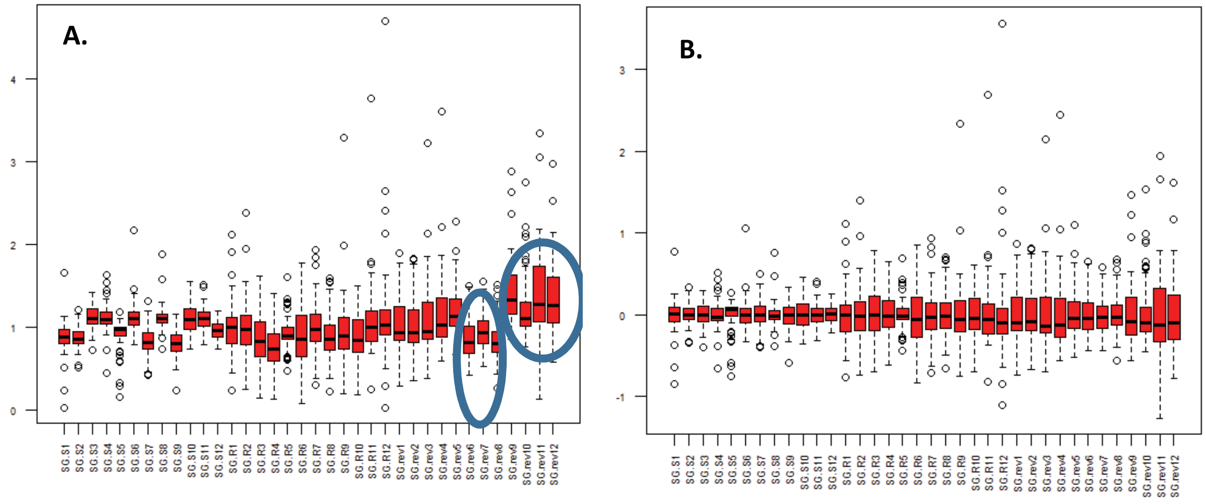

Figure S1. A box-plot (A) of the distribution of the normalized data for the SG series show that, despite the means being normalized, within each group the means are different and batch effects persist. To adjust for unequal means and batch effects within groups we centered the data (B) so all samples have the same means.
