# Supplementary material for: Intermediate-Type Vancomycin Resistance (VISA) in Genetically-Distinct Staphylococcus aureus Isolates Is Linked to Specific, Reversible Metabolic Alterations
Source: PLoS One. 2014 May 9;9(5):e97137. doi: 10.1371/journal.pone.0097137 (PMC4016254; doi:10.1371/journal.pone.0097137)
Supplement: Figure S2 — Box-plot of the distribution of normalized data for the JH series. Box plot (A) of the distribution of the normalized data for the JH series similarly shows that despite normalization, within each group the means are different. Box plot (B) shows the distribution after centering the data. (PDF) [file pone.0097137.s002.pdf]

A.

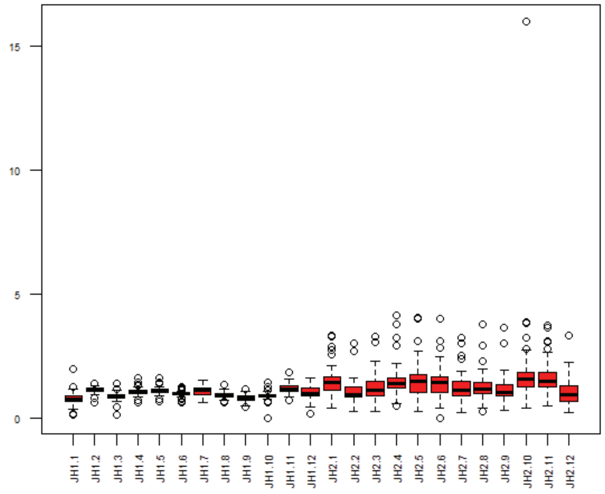

B.

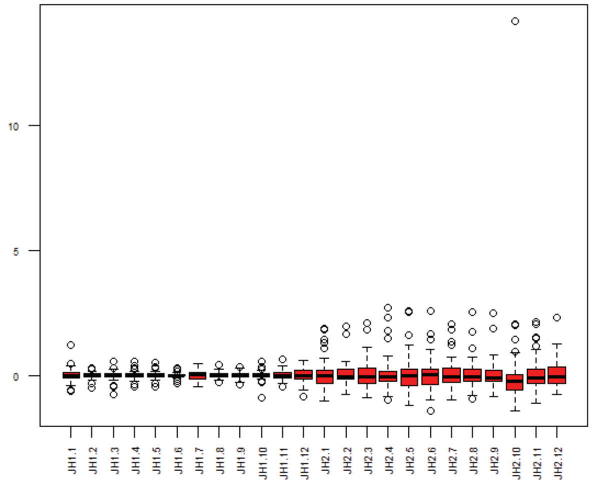

Figure S2. Box plot (A) of the distribution of the normalized data for the JH series similarly shows that despite normalization, within each group the means are different. Box plot (B) shows the distribution after centering the data.
