# Supplementary material for: Intermediate-Type Vancomycin Resistance (VISA) in Genetically-Distinct Staphylococcus aureus Isolates Is Linked to Specific, Reversible Metabolic Alterations
Source: PLoS One. 2014 May 9;9(5):e97137. doi: 10.1371/journal.pone.0097137 (PMC4016254; doi:10.1371/journal.pone.0097137)
Supplement: Figure S3 — Summary statistics for all analyses performed using hierarchical mixture modeling. (A) Displays the output summary statistics, including minimum, quartiles, maximum, mean, variance and coefficient of variance (CV) from the analysis of the 72 named metabolites in isolates JH1 (WT1-12) and JH2 (M1-12). Notice that the overall distributions of the metabolites are very similar across individuals and groups. (B) Show the same output summary statistics from analysis of the 72 named metabolites in isolates SG-S versus SG-R and SG-R versus SG-rev. (DOCX) [file pone.0097137.s003.docx]

Min. 1st Q. Median 3rd Q. Max. Mean Var.

**A.**

JH1.1 -0.6454251 -0.11460717 -0.0144754309 0.09961636 1.2026735 -3.007265e-17 0.06173319

JH1.2 -0.5053465 -0.04470267 0.0068426084 0.05399928 0.2666965 6.159977e-18 0.01216448

JH1.3 -0.7483187 -0.06061095 0.0287314220 0.07955920 0.5316245 1.619206e-17 0.02979168

JH1.4 -0.4446459 -0.06188905 0.0008738688 0.05751917 0.5514086 -7.246846e-17 0.02016861

JH1.5 -0.4610306 -0.06932040 -0.0040173367 0.05698473 0.5163721 -8.326673e-17 0.01979458

JH1.6 -0.3468808 -0.03043337 0.0007663088 0.03683276 0.2985215 3.855243e-17 0.01084072

JH1.7 -0.4789167 -0.13002881 0.0144799544 0.13306128 0.4532507 -7.401809e-17 0.03725359

JH1.8 -0.2966826 -0.06536741 0.0061367134 0.06469709 0.4133652 -1.540606e-18 0.01447248

JH1.9 -0.3693138 -0.07746507 -0.0007954708 0.08666235 0.3449979 2.620561e-17 0.01648777

JH1.10 -0.9183900 -0.04597484 0.0069336385 0.04978200 0.5299089 -1.696183e-17 0.02435458

JH1.11 -0.4598598 -0.10425164 -0.0232496960 0.09475839 0.6495320 -1.849755e-17 0.03054864

JH1.12 -0.8734669 -0.14239868 -0.0359479214 0.19545935 0.6021027 1.017749e-16 0.06704253

Min. 1st Q. Median 3rd Q. Max. Mean Var.

JH2.1 -1.0364074 -0.3060677 -0.035907772 0.19777949 1.859594 7.645696e-18 0.3332433

JH2.2 -0.7611689 -0.1544826 -0.066942186 0.24346618 1.972237 -1.094667e-16 0.2000247

JH2.3 -0.9135667 -0.3146540 -0.075195131 0.26397455 2.100460 -6.782889e-17 0.3026881

JH2.4 -0.9770593 -0.2505291 -0.083332637 0.15288071 2.690764 1.079203e-16 0.4200922

JH2.5 -1.2064313 -0.4185702 -0.001653978 0.26087747 2.567215 5.862541e-17 0.4736284

JH2.6 -1.4307886 -0.3871477 0.027427418 0.23319697 2.595743 -1.529600e-18 0.3836155

JH2.7 -0.9824809 -0.2959305 -0.068172219 0.27030486 2.046609 -2.619816e-17 0.2960439

JH2.8 -0.9457034 -0.2346110 -0.066501220 0.21303900 2.545607 -9.094179e-17 0.2915076

JH2.9 -0.8341252 -0.2206337 -0.089002859 0.21159316 2.486227 9.869214e-17 0.2799829

JH2.10 -1.4290523 -0.5559842 -0.240126868 0.01605072 14.204395 8.186103e-17 3.3144146

JH2.11 -1.1247911 -0.3403590 -0.102437099 0.23291736 2.148526 1.531177e-17 0.4129388

JH2.12 -0.7776755 -0.3036007 -0.055080031 0.31107375 2.321323 -8.326673e-17 0.2391799

**Min. 1st Q. Median 3rd Q. Max. Mean Var.**

**SG.S1 -0.8509605 -0.08482546 0.0001064356 0.08780047 0.7701137 5.088599e-17 0.03918565**

**B.**

**SG.S2 -0.3512268 -0.06451253 -0.0082907662 0.07440269 0.3389933 -4.009228e-17 0.01432059**

**SG.S3 -0.3950973 -0.08445969 -0.0080766505 0.10529621 0.2960275 -8.018842e-17 0.01951102**

**SG.S4 -0.6647368 -0.07510336 -0.0290389168 0.06501610 0.5156536 -3.079718e-18 0.02602686**

**SG.S5 -0.7553893 -0.01880039 0.0548144097 0.08639991 0.2636587 -6.159435e-18 0.03106677**

**SG.S6 -0.3273330 -0.09577426 -0.0095436541 0.06734789 1.0510193 -5.088607e-17 0.03175306**

**SG.S7 -0.4039260 -0.09086029 -0.0114823711 0.09732226 0.4905724 -5.241572e-17 0.02773316**

**SG.S8 -0.3836570 -0.06142271 -0.0136717204 0.04100803 0.7578893 6.156424e-18 0.02084880**

**SG.S9 -0.5857311 -0.11002070 -0.0134621486 0.08924412 0.3397354 -1.156619e-17 0.02815463**

**SG.S10 -0.3627515 -0.12511586 -0.0019587700 0.12140363 0.4542474 -3.391445e-17 0.02605497**

**SG.S11 -0.3162326 -0.09378728 -0.0118766995 0.07096088 0.3997543 0.000000e+00 0.01733782**

**SG.S12 -0.2238959 -0.07270313 0.0020741663 0.07745486 0.2461317 2.159256e-17 0.01169238**

**Min. 1st Q. Median 3rd Q. Max. Mean Var.**

**SG.R1 -0.7664081 -0.20702501 -0.004255764 0.10669949 1.1103982 4.472193e-17 0.09926174**

**SG.R2 -0.7397192 -0.18830538 -0.017334204 0.16288442 1.3937094 4.163336e-17 0.10894677**

**SG.R3 -0.6987726 -0.19511481 -0.006340941 0.22236921 0.7794724 2.317906e-18 0.09425818**

**SG.R4 -0.6240861 -0.15614567 -0.016200172 0.16951230 0.6515173 1.387779e-17 0.07233038**

**SG.R5 -0.4409608 -0.06244934 -0.021848040 0.07947480 0.6896077 4.548426e-17 0.03625417**

**SG.R6 -0.8432787 -0.27684135 -0.062616886 0.20984161 0.8518578 -4.618706e-18 0.11408442**

**SG.R7 -0.7095300 -0.18190539 -0.034669612 0.14611153 0.9278632 3.088564e-18 0.08690610**

**SG.R8 -0.6600685 -0.16169694 -0.024892141 0.13637262 0.7018938 -4.625802e-17 0.07190481**

**SG.R9 -0.7601795 -0.21155368 -0.061507115 0.15997561 2.3388184 4.701861e-17 0.15274231**

**SG.R10 -0.6960920 -0.17994282 -0.044601497 0.19989537 0.6074477 3.930967e-17 0.08203447**

**SG.R11 -0.8219333 -0.23738858 -0.065056402 0.12181986 2.6929914 -8.943049e-17 0.18140212**

**SG.R12 -1.1122395 -0.23333359 -0.108244065 0.06682117 3.5644887 -1.698809e-17 0.33217459**

**Min. 1st Q. Median 3rd Q. Max. Mean Var.**

**SG.rev1 -0.7396796 -0.1990065 -0.10687600 0.20495052 0.8584438 4.621224e-18 0.09153502**

**SG.rev2 -0.6700757 -0.1991365 -0.09196554 0.18665758 0.8084838 -5.395407e-17 0.08371404**

**SG.rev3 -0.6976777 -0.2273323 -0.13655406 0.21012702 2.1437163 -2.775558e-17 0.16319818**

**SG.rev4 -0.5603133 -0.2739402 -0.12904616 0.18697895 2.4407086 -4.007779e-17 0.20156777**

**SG.rev5 -0.5177492 -0.1602400 -0.05197098 0.15742220 1.0923859 9.098527e-17 0.08220737**

**SG.rev6 -0.4437467 -0.1717724 -0.04754463 0.14645922 0.6458581 1.079685e-17 0.05880449**

**SG.rev7 -0.4370044 -0.1680064 -0.03375231 0.10628066 0.5823301 5.396089e-17 0.04129914**

**SG.rev8 -0.5624192 -0.1346646 -0.02887235 0.11230204 0.6785824 -4.395480e-17 0.05616417**

**SG.rev9 -0.5619176 -0.2533950 -0.08465903 0.21108116 1.4616902 4.008348e-17 0.14952849**

**SG.rev10 -0.4623430 -0.2069414 -0.10878710 0.08531221 1.5300902 -2.931299e-17 0.12853577**

**SG.rev11 -1.2732177 -0.3397292 -0.12469583 0.31596433 1.9392207 1.851402e-17 0.26550128**

**SG.rev12 -0.7886612 -0.3046164 -0.09868998 0.23757539 1.6102271 -2.775558e-17 0.18443194**

**Figure S3:** **(A)** Displays the output summary statistics, including minimum, quartiles, maximum, mean, variance and coefficient of variance (CV) from the analysis of the 72 named metabolites in isolates JH1 (WT1-12) and JH2 (M1-12) . Notice that the overall distributions of the metabolites are very similar across individuals and groups. **(B)** Show the same output summary statistics from analysis of the 72 named metabolites in isolates SG-S versus SG-R and SG-R versus SG-rev**.**
