# Supplementary figures and images for: Intermediate-Type Vancomycin Resistance (VISA) in Genetically-Distinct Staphylococcus aureus Isolates Is Linked to Specific, Reversible Metabolic Alterations
Source: PLoS One. 2014 May 9;9(5):e97137. doi: 10.1371/journal.pone.0097137 (PMC4016254; doi:10.1371/journal.pone.0097137)

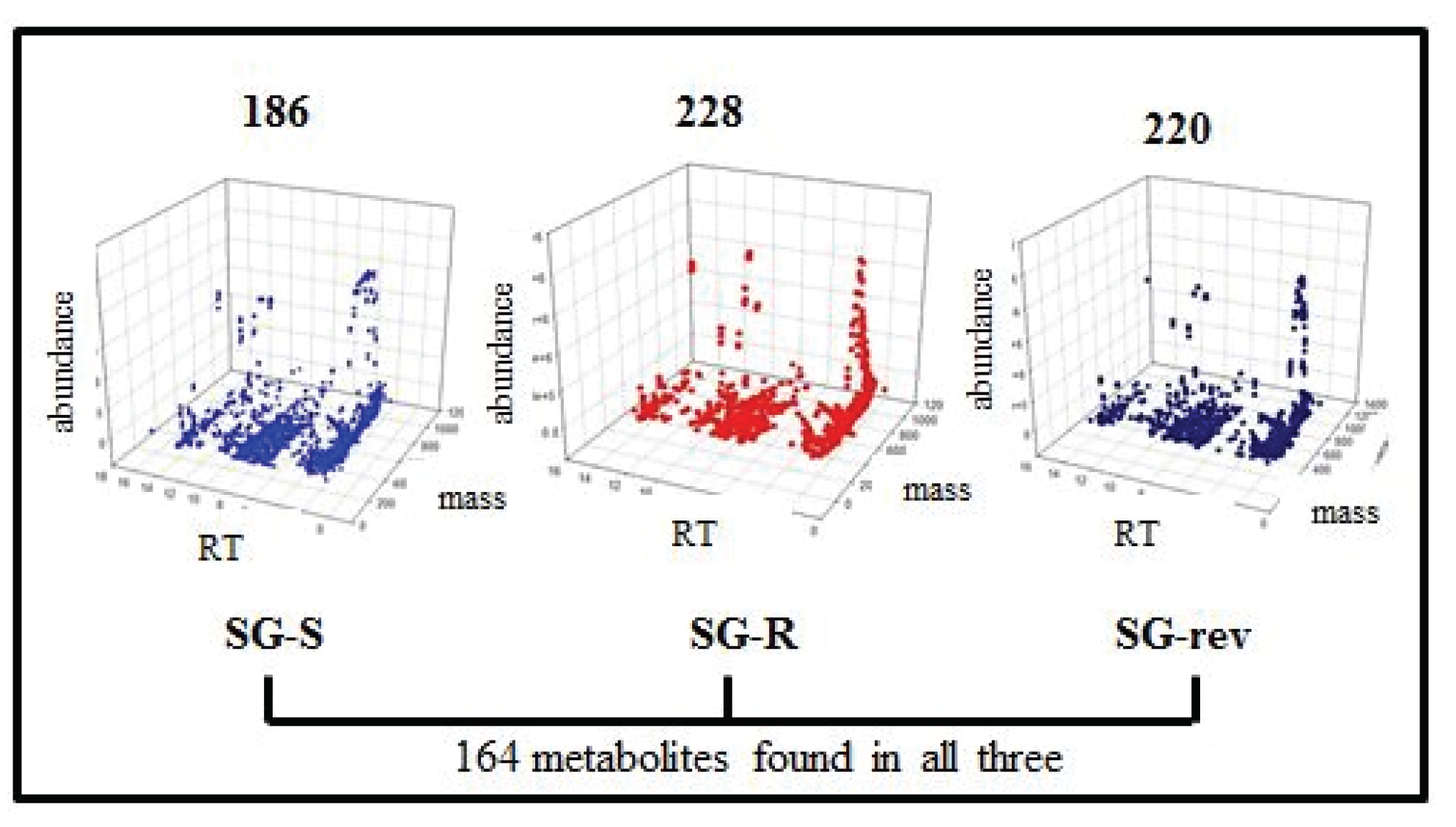

Supplement: Figure S4 — Metabolomic comparisons of isogenic VSSA (SGS, SG-rev, JH1) and VISA (SG-R, JH2) strains demonstrate unique and specific metabolic changes. Schematic of data output from a representative LC-MS analysis of each isolate. Data correspond to individual metabolites represented by individual points on a three dimensional axis where the x-axis denotes chromatographic retention time in minutes (RT), z-axis denotes accurate mass in atomic mass units (mass) and y-axis denotes peak height in total ion counts, which can be used as an estimate of abundance (abundance). 186 unique metabolites were detected and quantified in SG-S (blue dots), compared against 228 in SG-R (red dots) and 220 in SG-rev (dark blue dots). A total of 164 metabolites were common to all three isolates in three independent experiments. (TIF) [file pone.0097137.s004.tif]

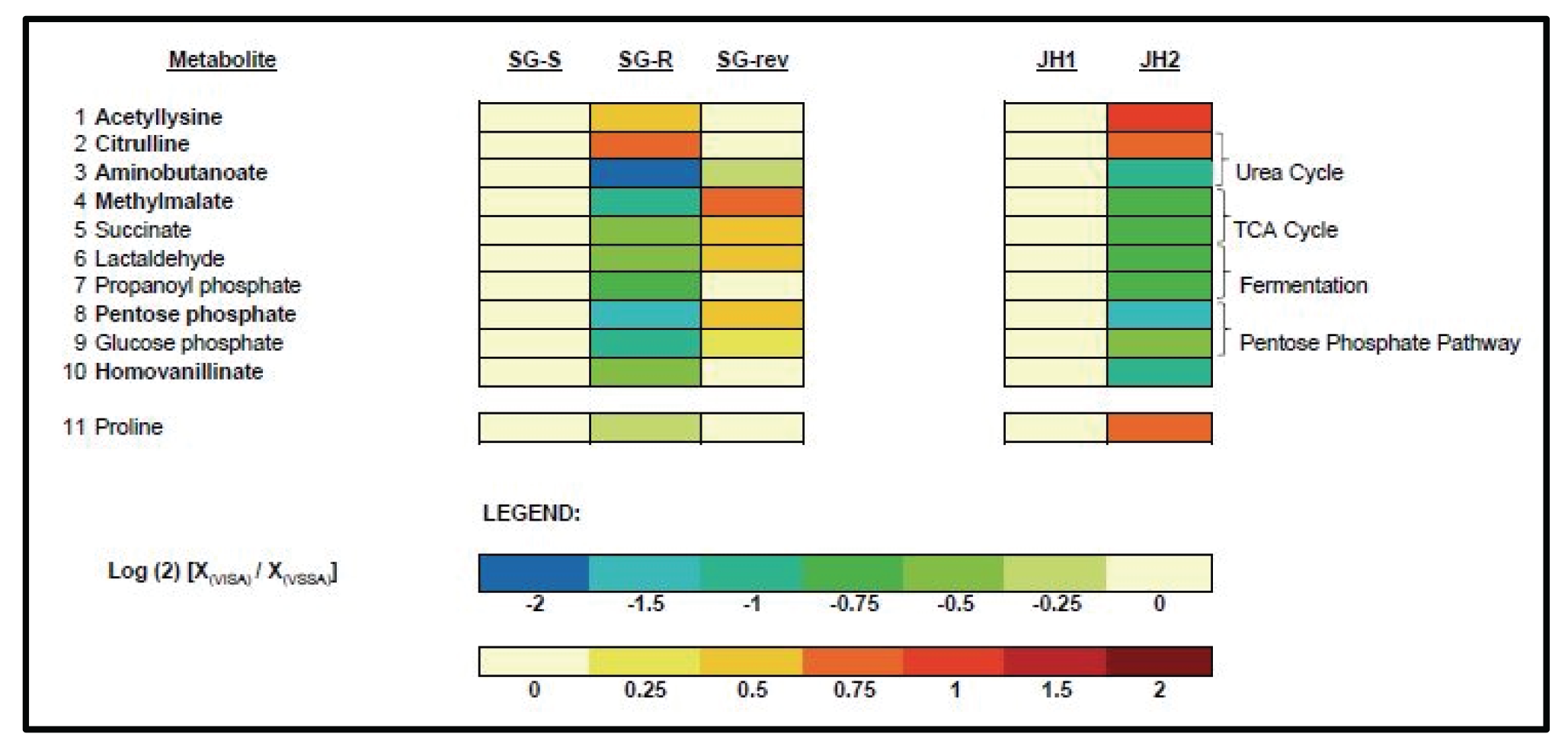

Supplement: Figure S5 — Heat map displaying the 11 metabolites whose abundance was significantly altered on SAM analysis (> 0.25-fold, FDR < 1) in the VISA, SG-R compared against the parent VSSA SG-S, and reversed directionality in SG-rev. Of these eleven metabolites, ten were significantly altered in a similar direction in the VISA isolate JH2 compared against its parent VSSA JH1. Changes abundance are indicated by color coding with red indicative of increases in mean intracellular abundance relative to the baseline (defined by the abundance in SG-S) and blue indicative of decreases in intracellular abundance on a log (2) scale. Bold font denotes the six metabolites whose abundance was also significantly altered in both VISA isolates on hierarchical mixture model analysis. (TIF) [file pone.0097137.s005.tif]
