# Supplementary material for: Intermediate-Type Vancomycin Resistance (VISA) in Genetically-Distinct Staphylococcus aureus Isolates Is Linked to Specific, Reversible Metabolic Alterations
Source: PLoS One. 2014 May 9;9(5):e97137. doi: 10.1371/journal.pone.0097137 (PMC4016254; doi:10.1371/journal.pone.0097137)
Supplement: Table S1 — List and characteristics of the 164 “core” metabolites (including 72 identified) in isolates SG-S, SG-R and SG-rev. Superscript (a) denotes Retention Time (RT), (b) denotes Positive Mode (POS) versus Negative Mode (NEG), (c) denotes Molecular Feature Extraction (MFE), which extracts chromatographic peaks by molecular features versus Find by Formula (FBF) which extracts peaks by chemical formula. Superscript (d) denotes metabolites confirmed by chemical standard (stnd). All other identifications are provisional identifications made by matching against a database of accurate mass-retention time pairs (mass-matching, MM), (e) denotes univariate statistical analysis of changes (> 0.25 – fold) in mean intracellular abundance by hierarchical modeling, adjusted by Benjamini-Hochberg procedure, (f) denotes bivariate analysis of changes (> 0.25 – fold) in intracellular abundance and variance by hierarchical modeling, adjusted by Benjamini-Hochberg procedure. Significance analysis of microarrays (SAM) analysis of changes in intracellular abundance (> 0.25-fold, FDR < 1%), significant metabolites denoted by an asterix (*). Bold font is used to indicate those metabolites whose abundance was altered in a similar fashion in the VISA isolate from both series (SG-R and JH2) and subsequently reversed in the revertant, SG-rev, as shown in Figure 4. (PDF) [file pone.0097137.s006.pdf]

Table S1. List and characteristics of the 164 "core" metabolites (including 72 identified) in isolates SG-S, SG-R and SG-rev.

							Abundance			Standard Deviation			Standard Error			p (SG-R versus SG-S)		(SG-R versus SG-S)	p (SG-R versus SG-rev)	
Name	Formula	Mass	RT[a]	Mode[b]	FbF/MFE[c]	MM/Stnd[d]	SG-S	SG-R	SG-rev	SG-S	SG-R	SG-Rev	SG-S	SG-R	SG-rev	Univariate[e]	Bivariate[f]	SAM	Univariate[e]	Bivariate[f]
Acetylaminobutanal	C6H11NO2	130.0863	7.5	POS	FbF	MM	1	0.923484	1.074218	0.154937	0.143972	0.352788	0.044726	0.041561	0.101841					
**Acetyllysine**	**C8H16N2O3**	**189.1234**	**1.3**	**POS**	**FbF**	**MM**	**1**	**1.469279**	**1.038538**	**0.347116**	**0.463426**	**0.57961**	**0.100204**	**0.13378**	**0.167319**	**< 0.01**	**< 0.01**	*****	**0.06**	**NS**
Acetylneuraminate	C11H19NO9	310.1133	2.5	POS	FbF	MM	1	0.98896	0.959795	0.156273	0.316849	0.284569	0.045112	0.091467	0.082148					
Acetylornithine	C7H14N2O3	175.1077	6	POS	FbF	MM	1	0.778985	0.876928	0.096294	0.069078	0.129621	0.027798	0.019941	0.037418			*		
Adenine	C5H5N5	136.0618	6.7	POS	FbF	MM	1	1.06411	0.966932	0.170441	0.218758	0.227917	0.049202	0.06315	0.065794					
Adenosine	C10H13N5O4	268.104	5.2	POS	FbF	MM	1	0.771609	0.777375	0.065643	0.069024	0.111362	0.018949	0.019925	0.032147			*		
Alanine	C3H7NO2	90.055	7.3	POS	FbF	standard	1	0.909987	0.856267	0.091726	0.112367	0.156532	0.026479	0.032438	0.045187					
Alpha-ketoglutarate	C5H6O5	145.0143	1	NEG	FbF	standard	1	0.839598	1.514367	0.231428	0.117305	0.358671	0.066807	0.033863	0.103539			*		
Aminoacrylate	C3H5NO2	88.0393	6	POS	FbF	MM	1	0.613919	0.844481	0.229383	0.136932	0.347323	0.066217	0.039529	0.100264			*		
Aminoadipate	C6H11NO4	162.0761	6	POS	FbF	MM	1	0.631352	0.820226	0.295345	1.497671	0.631546	0.085259	0.43234	0.182312	< 0.01	< 0.01		< 0.01	< 0.05
Aminobutyraldehyde	C4H9NO	88.07569	2	POS	FbF	MM	1	0.8463	1.022939	0.174853	0.191438	0.340486	0.050476	0.055263	0.09829					
Aminocyanobutanoic acid	C5H8N2O2	129.0659	7	POS	FbF	MM	1	0.645552	0.963332	0.168478	0.117299	0.221023	0.048635	0.033861	0.063804			*		
Aminocyclopropane carboxylic acid	C4H7NO2	102.055	6	POS	FbF	MM	1	0.740458	0.918946	0.195426	0.118032	0.303059	0.056415	0.034073	0.087486			*		
**Aminobutanoate**	**C4H9NO2**	**104.0706**	**6**	**POS**	**FbF***	**standard**	**1**	**0.217881**	**0.854082**	**0.147029**	**0.193553**	**0.117456**	**0.042443**	**0.055874**	**0.033907**	**< 0.01**	**< 0.01**	*****	**< 0.01**	**< 0.05**
Amino-oxopimelate	C7H11NO5	190.071	2.5	POS	FbF	MM	1	1.052571	1.000646	0.210538	0.232846	0.206761	0.060777	0.067217	0.059687					
Anthranilate	C7H7NO2	138.055	7.5	POS	FbF	MM	1	0.721654	0.83092	0.16143	0.229098	0.203264	0.046601	0.066135	0.058677					
Arginine	C6H14N4O2	175.119	13	POS	FbF	standard	1	0.925981	0.805117	0.052677	0.071807	0.156392	0.015207	0.020729	0.045147					
Aspargine	C4H8N2O3	133.0608	8	POS	FbF	standard	1	0.95353	0.83681	0.196112	0.167226	0.348611	0.056613	0.048274	0.100635					
Aspartate	C4H7NO4	134.0448	5.5	POS	FbF	standard	1	0.600656	0.782556	0.226349	0.135593	0.376746	0.065341	0.039142	0.108757			*		
Aspartate semialdehyde	C4H7NO3	118.0499	1.3	POS	FbF	MM	1	1.839666	2.487206	0.137784	0.480358	0.980309	0.039775	0.138668	0.282991	< 0.01	< 0.01	*	NS	NS
Carbamoyl aspartate	C5H8N2O5	177.0506	1.3	POS	FbF	MM	1	0.787375	0.980157	0.308004	0.175922	0.518533	0.088913	0.050784	0.149688					
Cis-aconitate	C6H6O6	173.0092	1	NEG	FbF	MM	1	1.056753	1.473807	0.122092	0.169239	0.356544	0.035245	0.048855	0.102926					
Citrate	C6H8O7	191.0197	1.4	NEG	FbF	standard	1	1.111278	1.265103	0.065977	0.133414	0.081643	0.019046	0.038513	0.023568					
**Citrulline**	**C6H13N3O3**	**176.103**	**9**	**POS**	**FbF**	**standard**	**1**	**1.633528**	**1.019183**	**0.194893**	**0.314853**	**0.203843**	**0.056261**	**0.09089**	**0.058844**	**< 0.01**	**< 0.01**	*****	**< 0.01**	**< 0.05**
d-ala-dala	C6H12N2O3	161.0921	7	POS	FbF	MM	1	0.865998	0.697402	0.266306	0.224254	0.237289	0.076876	0.064736	0.068499					
Deoxyadenosine	C10H13N5O3	252.1091	11	POS	FbF	MM	1	0.397526	0.411628	0.062861	0.314536	0.276458	0.018147	0.090799	0.079807					
Fumarate	C4H4O4	115.0037	1.2	NEG	FbF	standard	1	0.893414	1.273897	0.208019	0.142593	0.310989	0.06005	0.041163	0.089775					
Galactonate	C6H12O7	195.051	2.4	NEG	FbF	MM	1	0.864626	1.241979	0.203403	0.185369	0.205166	0.058717	0.053511	0.059226					
Glucose	C6H12O6	179.0561	2.2	NEG	FbF	standard	1	1.295632	1.42341	0.133169	0.137177	0.281805	0.038443	0.039599	0.08135	< 0.01	< 0.01	*	NS	NS
Glutamate	C5H9NO4	146.0459	6.4	NEG	FbF	standard	1	0.95128	1.332839	0.222281	0.166498	0.398066	0.064167	0.048064	0.114912					
Glutamine	C5H10N2O3	145.0619	8	NEG	FbF	standard	1	1.133111	1.586426	0.370147	0.559771	0.878739	0.106852	0.161592	0.25367					
Glycerate	C3H6O4	105.0193	2.3	NEG	FbF	MM	1	1.381826	1.906643	0.320244	0.259625	0.699394	0.092447	0.074947	0.201898	< 0.01	< 0.01	*	< 0.01	< 0.05
Glycine	C2H5NO2	76.0393	7	POS	FbF	standard	1	1.085894	0.815468	0.056524	0.152026	0.143558	0.016317	0.043886	0.041442					
Guanidonobutanoate	C5H11N3O2	130.0975	6.2	POS	FbF	MM	1	0.944683	0.749528	0.204608	0.220282	0.224656	0.059065	0.06359	0.064853					
Guanidinobutanal	C5H11N3O	146.0924	6	POS	FbF	MM	1	0.858653	0.917739	0.047039	0.239627	0.166736	0.013579	0.069174	0.048133					
Guanine	C5H5N5O	152.0567	6	POS	FbF	MM	1	0.915869	0.906286	0.116692	0.154992	0.155817	0.033686	0.044742	0.044981					
Guanosine	C10H13N5O5	284.099	3	POS	FbF	MM	1	0.652825	0.761439	0.088464	0.07925	0.109693	0.025537	0.022878	0.031666			*		
Hexose phosphate	C6H13O9P	259.0224	5.0-6.0	NEG	FbF	standard	1	0.465002	1.193006	0.153405	0.222532	0.322405	0.044284	0.06424	0.09307			*		
Histidine	C6H9N3O2	156.0768	12	POS	FbF	standard	1	1.026585	0.777617	0.245842	0.164832	0.265679	0.070969	0.047583	0.076695					
Homovanillin	C9H10O3	165.0557	1	NEG	FbF	MM	1	1.263675	1.057825	0.377098	0.356348	0.303722	0.108859	0.102869	0.087677					
**Homovanillinate**	**C9H10O4**	**181.0506**	**1.3**	**NEG**	**FbF**	**MM**	**1**	**0.670138**	**1.070788**	**0.250429**	**0.109402**	**0.423357**	**0.072293**	**0.031582**	**0.122213**	**< 0.05**	**NS**	*****	**0.06**	**NS**
Hypoxanthine	C5H4N4O	137.0458	2.5	POS	FbF	MM	1	1.073516	0.941069	0.130912	0.203771	0.181739	0.037791	0.058824	0.052464					
Lactaldehyde	C3H6O2	73.0295	1.3	NEG	FbF	MM	1	0.687728	1.311898	0.254423	0.096284	0.457926	0.073446	0.027795	0.132192			*		
Lactate	C3H6O3	89.02442	2.5	NEG	FbF	standard	1	1.242906	1.296777	0.143625	0.154184	0.287386	0.041461	0.044509	0.082961	<0.01	<0.01	*	NS	NS
Leucine	C6H13NO2	132.1019	6	POS	FbF	standard	1	0.924841	1.059153	0.115536	0.090431	0.102209	0.033352	0.026105	0.029505					
Lysine	C6H14N2O2	147.1128	12	POS	FbF	standard	1	0.770636	0.895513	0.29421	0.204277	0.166971	0.084931	0.05897	0.0482					
Malate	C4H6O5	133.0143	1.3	NEG	FbF	standard	1	0.98519	1.310312	0.177941	0.116564	0.39376	0.051367	0.033649	0.113669					
Methionine	C5H11NO2S	150.0583	5.6	POS	FbF	MM	1	0.773346	0.875352	0.087942	0.090049	0.111999	0.025387	0.025995	0.032331			*		
Methionine oxide	C5H11NO3S	166.0532	9.4	POS	FbF	MM	1	1.072472	0.968337	0.049095	0.099481	0.088942	0.014173	0.028718	0.025675					
Methylglyoxal	C3H4O2	71.01385	1.3	NEG	FbF	MM	1	0.343742	0.881903	0.262406	0.499159	0.340359	0.07575	0.144095	0.098253					
Methylthioadenosine	C11H15N5O3S	298.0968	4.5	POS	FbF	MM	1	1.087744	0.805887	0.061811	0.167935	0.149906	0.017843	0.048479	0.043274					
**Methymalate**	**C5H8O5**	**147.0299**	**1.3**	**NEG**	**FbF**	**MM**	**1**	**0.485834**	**1.565936**	**0.300271**	**0.17356**	**0.419497**	**0.086681**	**0.050102**	**0.121098**	**<0.01**	**<0.01**	*****	**<0.01**	**<0.01**
Ornithine	C5H12N2O2	133.0972	12	POS	FbF	standard	1	1.163482	0.796735	0.328578	0.300087	0.295385	0.094852	0.086628	0.08527					
Oxoisocapriopoic acid	C6H10O3	129.0557	6	NEG	FbF	MM	1	1.166124	1.706392	0.246722	0.161477	0.334154	0.071223	0.046614	0.096462					
Oxoproline	C5H7NO3	130.0499	1.5	POS	FbF	MM	1	0.786362	0.657382	0.539843	0.504622	0.798848	0.155839	0.145672	0.230607					
**Pentose phosphate**	**C5H11O8P**	**229.0119**	**4.3**	**NEG**	**FbF**	**MM**	**1**	**0.356736**	**1.37736**	**0.288669**	**0.219981**	**0.572849**	**0.083332**	**0.063503**	**0.165367**	**<0.01**	**<0.01**	*****	**<0.01**	**<0.01**
Phenylalanine	C9H11NO2	166.0863	5.5	POS	FbF	standard	1	0.867046	1.076365	0.151568	0.088381	0.155846	0.043754	0.025513	0.044989					
Phenylpyruvate	C9H8O3	163.0401	1.2	NEG	FbF	MM	1	0.701159	1.459753	0.262198	0.15677	0.370934	0.07569	0.045256	0.107079					
Proline	C5H9NO2	116.0706	8	POS	FbF	standard	1	0.801802	0.995126	0.168488	0.107576	0.209467	0.048638	0.031054	0.060468			*		
Propanoyl phosphate	C3H7O5P	152.9958	2.7	NEG	FbF	MM	1	0.644955	0.989407	0.117933	0.125353	0.214964	0.034044	0.036186	0.062055			*		
Propynoate	C3H2O2	68.9982	0.5	NEG	FbF	MM	1	0.905836	1.318814	0.213036	0.232603	0.271331	0.061498	0.067147	0.078327					
Pyruvate	C3H4O3	87.00877	1.5	NEG	FbF	standard	1	1.261675	1.397717	0.149393	0.1444	0.197326	0.043126	0.041685	0.056963	< 0.01	< 0.01	*	NS	NS
Serine	C3H7NO3	106.0499	7	POS	FbF	MM	1	1.031986	0.86434	0.044747	0.164958	0.130794	0.012917	0.047619	0.037757					
Succinate semialdehyde	C4H6O3	101.0244	1.3	NEG	FbF	MM	1	1.078008	1.810041	0.207965	0.206952	0.43375	0.060034	0.059742	0.125213					

Succinic acid	C4H6O4	117.0193	1	NEG	FbF	standard	1	0.664867	1.333608	0.263923	0.102556	0.513289	0.076188	0.029605	0.148174			*		
Succinylarginine	C10H18N4O5	275.135	9	POS	FbF	MM	1	0.809202	0.828366	0.166337	0.106764	0.288891	0.048017	0.03082	0.083396					
Tartronate semialdehyde	C3H4O4	103.0037	1.2	NEG	FbF	MM	1	1.027123	1.387024	0.134233	0.244037	0.429901	0.03875	0.070447	0.124102					
Threonine	C4H9NO3	120.0655	6.7	POS	FbF	MM	1	1.181481	0.886601	0.189266	0.156777	0.201309	0.054636	0.045258	0.058113					
Tryptophan	C11H12N2O2	205.0972	5	POS	FbF	standard	1	0.824303	0.866012	0.041526	0.114634	0.096432	0.011988	0.033092	0.027837					
Tyramine	C8H11NO	138.0913	5.7	POS	FbF	MM	1	0.75862	0.887078	0.062517	0.259008	0.12814	0.018047	0.074769	0.036991					
Tyrosine	C9H11NO3	182.0812	5.5	POS	FbF	standard	1	0.680746	0.911988	0.137991	0.107538	0.143568	0.039834	0.031044	0.041444			*		
Valine	C5H11NO2	118.0863	6	POS	FbF	standard	1	0.880527	1.100558	0.159041	0.11347	0.231039	0.045911	0.032756	0.066695					
1		66.0152	12.725	POS	MFE	NA	1	0.892013	0.977816	0.171358	0.223051	0.244133	0.049467	0.064389	0.070475					
2		71.0744	6.663	POS	MFE	NA	1	0.851316	0.902271	0.203098	0.06992	0.222442	0.058629	0.020184	0.064213					
3		83.0377	6.434	POS	MFE	NA	1	0.735646	0.918944	0.187495	0.227299	0.214732	0.054125	0.065616	0.061988	< 0.01	<0.05		< 0.01	NS
4		83.0738	12.352	POS	MFE	NA	1	0.804947	0.901519	0.082864	0.076274	0.149151	0.023921	0.022018	0.043056					
5		85.0529	2.094	POS	MFE	NA	1	0.975789	0.95405	0.118569	0.151995	0.157035	0.034228	0.043877	0.045332					
6		85.0896	6.106	POS	MFE	NA	1	0.873986	0.938447	0.22579	0.208135	0.192755	0.06518	0.060083	0.055644					
7		89.048	7.364	POS	MFE	NA	1	0.912598	0.860174	0.089802	0.105581	0.158416	0.025924	0.030479	0.045731					
8		99.0683	2.27	POS	MFE	NA	1	0.940628	0.914287	0.07085	0.092803	0.123708	0.020453	0.02679	0.035711					
9		103.063	7.752	POS	MFE	NA	1	0.069318	0.042194	0.152675	0.239174	0.466065	0.044074	0.069044	0.134541	< 0.01	<0.01		< 0.01	<0.01
10		113.0584	8.008	POS	MFE	NA	1	0.851477	0.888303	0.154883	0.12515	0.147738	0.044711	0.036128	0.042648					
11		119.0728	5.777	POS	MFE	NA	1	0.907157	0.917748	0.061804	0.087924	0.092363	0.017841	0.025382	0.026663					
12		131.0612	7.415	POS	MFE	NA	1	1.173877	1.852636	0.29309	0.194469	0.431541	0.084608	0.056138	0.124575					
13		196.1218	1.689	POS	MFE	NA	1	0.801772	0.845062	0.061674	0.104379	0.066485	0.017804	0.030132	0.019193					
14		197.0351	2.597	POS	MFE	NA	1	0.663496	0.845054	0.18351	0.132501	0.263977	0.052975	0.03825	0.076204	< 0.01	NS		NS	NS
15		211.0949	2.723	POS	MFE	NA	1	0.990226	0.843131	0.081467	0.102704	0.107159	0.023518	0.029648	0.030934					
16		226.1213	1.165	POS	MFE	NA	1	0.835555	0.861482	0.067988	0.135534	0.091036	0.019626	0.039125	0.02628					
17		228.0889	1.659	POS	MFE	NA	1	0.829954	0.873634	0.055243	0.102778	0.058403	0.015947	0.02967	0.01686					
18		244.1223	1.586	POS	MFE	NA	1	0.819177	0.841755	0.051348	0.103415	0.073989	0.014823	0.029853	0.021359					
19		256.1419	9.229	POS	MFE	NA	1	0.972181	1.113492	0.322666	0.338237	0.616173	0.093146	0.097641	0.177874					
20		260.1184	1.613	POS	MFE	NA	1	0.812918	0.883862	0.084753	0.104356	0.091939	0.024466	0.030125	0.02654					
21		264.078	5.869	POS	MFE	NA	1	0.613774	0.805991	0.230708	0.160606	0.37801	0.0666	0.046363	0.109122	< 0.01	NS		< 0.05	NS
22		311.1806	1.658	POS	MFE	NA	1	0.956538	0.835644	0.180611	0.176806	0.153903	0.052138	0.051039	0.044428					
23		314.1688	1.243	POS	MFE	NA	1	0.737119	0.897641	0.129674	0.103877	0.201229	0.037434	0.029987	0.05809					
24		338.2473	1.162	POS	MFE	NA	1	0.952889	0.854139	0.071061	0.125627	0.106662	0.020514	0.036265	0.030791					
25		355.2752	1.159	POS	MFE	NA	1	0.725559	0.753115	0.162895	0.134057	0.110058	0.047024	0.038699	0.031771	< 0.05	NS		NS	NS
26		382.2741	1.185	POS	MFE	NA	1	0.933194	0.846041	0.082697	0.122682	0.132448	0.023872	0.035415	0.038235					
27		384.2392	7.234	POS	MFE	NA	1	1.041311	0.830726	0.089804	0.13383	0.112521	0.025924	0.038633	0.032482					
28		389.1935	9.263	POS	MFE	NA	1	0.695412	0.653872	0.165334	0.142572	0.356578	0.047728	0.041157	0.102935					
29		397.2809	1.144	POS	MFE	NA	1	0.855459	0.89576	0.083766	0.190855	0.23276	0.024181	0.055095	0.067192					
30		399.3012	1.178	POS	MFE	NA	1	0.74911	0.779743	0.134055	0.107813	0.115326	0.038698	0.031123	0.033292	< 0.05	NS		NS	NS
31		411.2693	1.855	POS	MFE	NA	1	0.983063	0.833168	0.618923	0.263316	0.276262	0.178668	0.076013	0.07975					
32		411.7716	1.851	POS	MFE	NA	1	0.963284	0.842071	0.113259	0.129709	0.126854	0.032695	0.037444	0.03662					
33		420.2272	1.163	POS	MFE	NA	1	0.757303	0.854127	0.293119	0.147745	0.281789	0.084616	0.04265	0.081345					
34		426.2965	1.197	POS	MFE	NA	1	0.910413	0.98383	0.105173	0.130527	0.107329	0.030361	0.03768	0.030983					
35		433.2844	1.983	POS	MFE	NA	1	1.00631	0.770418	0.147525	0.156978	0.178088	0.042587	0.045316	0.05141					
36		433.7792	1.978	POS	MFE	NA	1	0.964028	0.867782	0.135806	0.152558	0.14158	0.039204	0.04404	0.040871					
37		441.2096	13.419	POS	MFE	NA	1	1.024973	0.787284	0.071967	0.059656	0.115815	0.020775	0.017221	0.033433					
38		443.3232	1.193	POS	MFE	NA	1	0.751712	0.799471	0.147414	0.117901	0.126139	0.042555	0.034035	0.036413	< 0.05	NS		NS	NS
39		455.2826	2.121	POS	MFE	NA	1	1.241114	0.85358	0.094031	0.206361	0.167124	0.027145	0.059571	0.048244					
40		460.281	12.611	POS	MFE	NA	1	1.002274	0.870182	0.047015	0.103697	0.081391	0.013572	0.029935	0.023495					
41		474.2182	6.361	POS	MFE	NA	1	1.000998	0.899475	0.051675	0.127973	0.081146	0.014917	0.036943	0.023425					
42		485.3104	2.148	POS	MFE	NA	1	0.379258	0.837661	0.170313	0.295155	0.156189	0.049165	0.085204	0.045088	< 0.01	< 0.01		< 0.01	NS
43		485.8201	2.347	POS	MFE	NA	1	0.890059	0.797215	0.092132	0.099993	0.07567	0.026596	0.028865	0.021844					
44		486.3167	2.335	POS	MFE	NA	1	1.016202	0.797592	0.083896	0.115909	0.113949	0.024219	0.03346	0.032894					
45		487.354	1.239	POS	MFE	NA	1	0.735629	0.793954	0.176735	0.114204	0.168831	0.051019	0.032968	0.048737	< 0.05	NS		NS	NS
46		524.261	3.601	POS	MFE	NA	1	1.319172	1.147413	0.097385	0.172231	0.211971	0.028113	0.049719	0.061191					
47		525.8088	12.552	POS	MFE	NA	1	1.024177	0.817408	0.08566	0.092469	0.090084	0.024728	0.026693	0.026005					
48		529.8452	2.776	POS	MFE	NA	1	1.042416	0.762934	0.138597	0.133327	0.140015	0.040009	0.038488	0.040419					
49		531.3806	1.456	POS	MFE	NA	1	0.719812	0.81806	0.172723	0.148957	0.170714	0.049861	0.043	0.049281	< 0.05	NS		NS	NS
50		543.3401	3.0852	POS	MFE	NA	1	1.150858	0.986496	0.15969	0.175499	0.230144	0.046099	0.050662	0.066437					
51		551.8591	3.076	POS	MFE	NA	1	1.064367	0.76996	0.129711	0.263385	0.244075	0.037444	0.076033	0.070458					
52		561.814	7.62	POS	MFE	NA	1	1.04878	0.752023	0.135454	0.130177	0.122694	0.039102	0.037579	0.035419					
53		567.834	12.674	POS	MFE	NA	1	0.962908	0.726865	0.086944	0.099617	0.120728	0.025099	0.028757	0.034851					
54		575.407	1.324	POS	MFE	NA	1	0.718877	0.843331	0.18102	0.116685	0.182791	0.052256	0.033684	0.052767	< 0.05	NS		NS	NS
55		614.2758	10.058	POS	MFE	NA	1	1.026208	0.80901	0.090932	0.09461	0.102749	0.02625	0.027312	0.029661					
56		619.4338	1.368	POS	MFE	NA	1	0.745046	0.860752	0.181584	0.119625	0.166853	0.052419	0.034533	0.048166	< 0.05	NS		NS	NS
57		707.482	1.375	POS	MFE	NA	1	0.866596	0.872807	0.112931	0.087009	0.097555	0.0326	0.025117	0.028162					
58		728.4138	1.615	POS	MFE	NA	1	0.781489	0.870372	0.183426	0.14586	0.178789	0.052951	0.042106	0.051612					
59		820.4329	2.365	POS	MFE	NA	1	1.00274	0.865155	0.068974	0.102395	0.096002	0.019911	0.029559	0.027714					
60		839.5653	1.861	POS	MFE	NA	1	0.807551	0.814501	0.090856	0.083813	0.094544	0.026228	0.024195	0.027293					
61		90.0335	1.38	NEG	MFE	NA	1	1.021514	0.722897	0.147867	0.14654	0.265336	0.042686	0.042302	0.076596					

62		146.1082	12.327	NEG	MFE	NA	1	0.973864	1.448123	0.148837	0.122407	0.425901	0.042966	0.035336	0.122947					
63		163.0868	9.343	NEG	MFE	NA	1	1.101436	1.504103	0.248591	0.208865	0.336754	0.071762	0.060294	0.097213					
64		165.0792	5.752	NEG	MFE	NA	1	1.08872	1.401074	0.146028	0.129362	0.226143	0.042155	0.037344	0.065282					
65		174.1149	12.335	NEG	MFE	NA	1	1.153918	1.240428	0.076583	0.074692	0.162966	0.022108	0.021562	0.047044					
66		192.031	1.365	NEG	MFE	NA	1	1.141617	1.267752	0.070298	0.085552	0.071118	0.020293	0.024697	0.02053					
67		197.0388	2.593	NEG	MFE	NA	1	0.962157	1.373168	0.203393	0.136055	0.355784	0.058714	0.039276	0.102706					
68		200.0846	1.907	NEG	MFE	NA	1	1.051335	1.53068	0.170978	0.155582	0.223493	0.049357	0.044913	0.064517					
69		216.0402	2.665	NEG	MFE	NA	1	1.364897	1.524521	0.115818	0.155733	0.124763	0.033434	0.044956	0.036016	< 0.05	NS		NS	NS
70		226.0719	2.655	NEG	MFE	NA	1	1.295424	1.467279	0.128604	0.144155	0.180562	0.037125	0.041614	0.052124	< 0.05	NS		NS	NS
71		264.0829	5.858	NEG	MFE	NA	1	0.95	1.332495	0.199118	0.131588	0.410739	0.05748	0.037986	0.11857					
72		270.1329	0.984	NEG	MFE	NA	1	1.139882	1.438837	0.134992	0.072963	0.164726	0.038969	0.021062	0.047552					
73		278.0441	2.661	NEG	MFE	NA	1	1.447829	1.373572	0.08563	0.218562	0.149046	0.024719	0.063093	0.043026	< 0.01	NS		NS	NS
74		284.1486	0.987	NEG	MFE	NA	1	1.221824	1.444242	0.140058	0.083269	0.163413	0.040431	0.024038	0.047173					
75		298.1643	1.002	NEG	MFE	NA	1	1.210572	1.51102	0.170307	0.070378	0.191157	0.049163	0.020317	0.055182					
76		310.1623	1.024	NEG	MFE	NA	1	1.063061	1.375598	0.120753	0.095173	0.141494	0.034859	0.027474	0.040846					
77		312.1804	1.021	NEG	MFE	NA	1	1.143124	1.47922	0.152346	0.083013	0.165476	0.043978	0.023964	0.047769					
78		316.1292	1.261	NEG	MFE	NA	1	1.339512	1.443942	0.107464	0.12642	0.169813	0.031022	0.036494	0.049021	< 0.05	NS		NS	NS
79		324.1785	1.035	NEG	MFE	NA	1	1.143219	1.50892	0.13453	0.098907	0.173499	0.038835	0.028552	0.050085					
80		326.1967	1.032	NEG	MFE	NA	1	1.14074	1.545177	0.162492	0.104781	0.191662	0.046907	0.030248	0.055328					
81		340.2113	1.062	NEG	MFE	NA	1	1.08631	1.52628	0.196915	0.135167	0.214707	0.056845	0.039019	0.061981					
82		354.227	1.08	NEG	MFE	NA	1	1.105397	1.603331	0.182278	0.16275	0.234156	0.052619	0.046982	0.067595					
83		368.246	1.109	NEG	MFE	NA	1	1.120895	1.566269	0.171344	0.119813	0.212066	0.049463	0.034587	0.061218					
84		388.1328	4.38	NEG	MFE	NA	1	1.262715	1.356738	0.111865	0.217586	0.257565	0.032293	0.062812	0.074353					
85		417.1481	1.382	NEG	MFE	NA	1	1.13402	1.386612	0.069585	0.09826	0.19569	0.020088	0.028365	0.056491					
86		454.2199	2.032	NEG	MFE	NA	1	1.22595	1.536416	0.145209	0.190304	0.21128	0.041918	0.054936	0.060991					
87		474.3011	1.306	NEG	MFE	NA	1	0.738427	1.521059	0.315137	0.223022	0.577598	0.090972	0.064381	0.166738					
88		552.1982	2.053	NEG	MFE	NA	1	1.083186	1.346662	0.091516	0.076091	0.128447	0.026418	0.021966	0.037079					
89		575.3608	12.597	NEG	MFE	NA	1	1.406711	1.411404	0.097683	0.093743	0.12505	0.028199	0.027061	0.036099	<0.05	NS		NS	NS
90		726.4559	1.627	NEG	MFE	NA	1	1.342339	1.38584	0.147084	0.158124	0.215305	0.04246	0.045647	0.062153	< 0.05	NS		NS	NS
91		775.3619	1.901	NEG	MFE	NA	1	1.049325	1.456682	0.166437	0.097756	0.21319	0.048046	0.02822	0.061543					
92		814.5114	1.759	NEG	MFE	NA	1	1.457814	1.286446	0.150377	0.149674	0.189839	0.04341	0.043207	0.054802	< 0.05	NS		NS	NS

[a] Retention Time (RT)

[b] Positive Mode (POS) versus Negative Mode (NEG)

[c] Molecular Feature Extraction (MFE) extracts chromatographic peaks by molecular features versus Find by Formula (FBF) which extracts peaks by chemical formula.

[d] denotes metabolites confirmed by chemical standard (stnd). All other identifications are provisional identifications made by matching against a database of accurate mass-retention time pairs (mass-matching, MM).

[e] Univariate statististical analysis of changes (> 0.25-fold) in mean intracellular abundance by heirarchical modeling, adjusted by Benjamini-Hochberg procedure.

[f] Bivariate analysis of changes in intracellular abundance and variance by heirarchical modeling, adjusted by Benjamini-Hochberg procedure

Significance Analysis of Microarrays (SAM) analysis of changes in intracellular abundance (> 0.25-fold, FDR < 1%), significant metabolites denoted by an asterix (*)

Bold font is used to indicate those metabolites whose abundance was altered in a similar fashion in the VISA isolate from both series (SG-R and JH2) and subsequently reversed in the revertant, SG-rev, as shown in Figure 4.
