# Supplementary material for: Intermediate-Type Vancomycin Resistance (VISA) in Genetically-Distinct Staphylococcus aureus Isolates Is Linked to Specific, Reversible Metabolic Alterations
Source: PLoS One. 2014 May 9;9(5):e97137. doi: 10.1371/journal.pone.0097137 (PMC4016254; doi:10.1371/journal.pone.0097137)
Supplement: Table S2 — List and characteristics of the 72 identified metabolites for isolates JH1 and JH2. Superscript (a) denotes Retention Time (RT), (b) denotes Positive Mode (POS) versus Negative Mode (NEG), (c) denotes Molecular Feature Extraction (MFE), which extracts chromatographic peaks by molecular features versus Find by Formula (FBF) which extracts peaks by chemical formula. Superscript (d) denotes metabolites confirmed by chemical standard (stnd). All other identifications are provisional identifications made by matching against a database of accurate mass-retention time pairs (mass-matching, MM), (e) denotes univariate statistical analysis of changes (> 0.25 – fold) in mean intracellular abundance by hierarchical modeling, adjusted by Benjamini-Hochberg procedure, (f) denotes bivariate analysis of changes (> 0.25 – fold) in intracellular abundance and variance by hierarchical modeling, adjusted by Benjamini-Hochberg procedure. Significance analysis of microarrays (SAM) analysis of changes in intracellular abundance (> 0.25-fold, FDR < 1%), significant metabolites denoted by an asterix (*). Bold font is used to indicate those metabolites whose abundance was altered in a similar fashion in the VISA isolate from both series (SG-R and JH2) and subsequently reversed in the revertant, SG-rev, as shown in Figure 4. (PDF) [file pone.0097137.s007.pdf]

Table S2. List and characteristics of the 72 identified metabolites for isolates JH1 and JH2.

							Abundance		Standard Deviation		Standard Error		*p* (JH2 versus JH1)		(JH2 versus JH1)
**Name**	**Formula**	**Mass**	**RT[a]**	**Mode[b]**	**FbF/MFE[c]**	**MM/Stnd[d]**	**JH1**	**JH2**	**JH1**	**JH2**	**JH1**	**JH2**	**Univariate[e]**	**Bivariate[f]**	**SAM**
Acetylaminobutanal	C6H11NO2	130.0863	7.5	POS	FbF	MM	1	2.82135	0.375663	1.093531	0.108444	0.315675	< 0.01	< 0.01	*
**Acetyllysine**	**C8H16N2O3**	**189.1234**	**1.3**	**POS**	**FbF**	**MM**	**1**	**2.072416**	**0.4083**	**1.112968**	**0.117866**	**0.321286**	**< 0.01**	**< 0.01**	*
Acetylneuraminate	C11H19NO9	310.1133	2.5	POS	FbF	MM	1	1.6999	0.130274	0.149844	0.037607	0.043256	< 0.05	< 0.05	*
Acetylornithine	C7H14N2O3	175.1077	6	POS	FbF	MM	1	1.409221	0.132918	0.153497	0.03837	0.044311			*
Adenine	C5H5N5	136.0618	6.7	POS	FbF	MM	1	3.253274	0.29467	0.553344	0.085064	0.159737	< 0.01	< 0.01	*
Adenosine	C10H13N5O4	268.104	5.2	POS	FbF	MM	1	1.17642	0.290578	0.501087	0.083883	0.144651			
Alanine	C3H7NO2	90.055	7.3	POS	FbF	standard	1	1.362019	0.138699	0.191234	0.040039	0.055205			*
Alpha-ketoglutarate	C5H6O5	145.0143	1	NEG	FbF	standard	1	1.395265	0.264082	0.424138	0.076234	0.122438			*
Aminoacrylate	C3H5NO2	88.0393	6	POS	FbF	MM	1	2.337199	0.265501	4.324602	0.076644	1.248405			
Aminoadipate	C6H11NO4	162.0761	6	POS	FbF	MM	1	1.710203	0.235715	0.645655	0.068045	0.186384	< 0.05		*
Aminobutyraldehyde	C4H9NO	88.07569	2	POS	FbF	MM	1	1.240773	0.158546	0.22271	0.045768	0.064291			*
Aminocyanobutanoic acid	C5H8N2O2	129.0659	7	POS	FbF	MM	1	1.153669	0.448017	0.346179	0.129331	0.099933			
Aminocyclopropane carboxylic acid	C4H7NO2	102.055	6	POS	FbF	MM	1	1.343845	0.210345	0.408623	0.060721	0.117959			
**Aminobutanoate**	**C4H9NO2**	**104.0706**	**6**	**POS**	**FbF***	**standard**	**1**	**0.456411**	**0.114785**	**0.071165**	**0.033136**	**0.020544**	**< 0.01**	**< 0.01**	*
Amino-oxopimelate	C7H11NO5	190.071	2.5	POS	FbF	MM	1	1.114464	0.330517	0.475999	0.095412	0.137409			
Anthranilate	C7H7NO2	138.055	7.5	POS	FbF	MM	1	0.817931	0.16976	0.2408	0.049006	0.069513	< 0.01	< 0.05	*
Arginine	C6H14N4O2	175.119	13	POS	FbF	standard	1	1.580176	0.170935	0.187684	0.049345	0.05418			*
Aspargine	C4H8N2O3	133.0608	8	POS	FbF	standard	1	1.459797	0.113681	0.24106	0.032817	0.069588			*
Aspartate	C4H7NO4	134.0448	5.5	POS	FbF	standard	1	1.213347	0.213009	0.300609	0.06149	0.086778			
Aspartate semialdehyde	C4H7NO3	118.0499	1.3	POS	FbF	MM	1	1.380308	0.14518	0.333151	0.04191	0.096172			*
Carbamoyl aspartate	C5H8N2O5	177.0506	1.3	POS	FbF	MM	1	1.430415	0.333617	0.604805	0.096307	0.174592			
Cis-aconitate	C6H6O6	173.0092	1	NEG	FbF	MM	1	1.338885	0.08703	0.276891	0.025123	0.079931			*
Citrate	C6H8O7	191.0197	1.4	NEG	FbF	standard	1	1.599105	0.059099	0.271666	0.01706	0.078423			*
**Citrulline**	**C6H13N3O3**	**176.103**	**9**	**POS**	**FbF**	**standard**	**1**	**1.681761**	**0.124049**	**0.380735**	**0.03581**	**0.109909**	**< 0.05**		*
d-ala-dala	C6H12N2O3	161.0921	7	POS	FbF	MM	1	1.835221	0.190114	0.466255	0.054881	0.134596	< 0.01	< 0.01	*
Deoxyadenosine	C10H13N5O3	252.1091	11	POS	FbF	MM	1	1.264649	0.143013	0.112243	0.041284	0.032402			*
Fumarate	C4H4O4	115.0037	1.2	NEG	FbF	standard	1	1.1968	0.180754	0.253797	0.052179	0.073265			
Galactonate	C6H12O7	195.051	2.4	NEG	FbF	MM	1	1.00468	0.071579	0.105715	0.020663	0.030517			
Glucose	C6H12O6	179.0561	2.2	NEG	FbF	standard	1	1.531567	0.11218	0.232003	0.032384	0.066973			*
Glutamate	C5H9NO4	146.0459	6.4	NEG	FbF	standard	1	1.408517	0.177456	0.361533	0.051227	0.104366			*
Glutamine	C5H10N2O3	145.0619	8	NEG	FbF	standard	1	1.827655	0.171655	0.477837	0.049553	0.13794	< 0.01	< 0.05	*
Glycerate	C3H6O4	105.0193	2.3	NEG	FbF	MM	1	1.852234	0.11987	0.291487	0.034604	0.084145	< 0.01	< 0.01	*
Glycine	C2H5NO2	76.0393	7	POS	FbF	standard	1	1.977622	0.643819	1.057298	0.185854	0.305216	< 0.01	< 0.01	*
Guanidonobutanoate	C5H11N3O2	130.0975	6.2	POS	FbF	MM	1	1.169129	0.446348	0.616919	0.12885	0.178089			
Guanidinobutanal	C5H11N3O	146.0924	6	POS	FbF	MM	1	1.049839	0.114845	0.110614	0.033153	0.031931			
Guanine	C5H5N5O	152.0567	6	POS	FbF	MM	1	0.893004	0.135959	0.178644	0.039248	0.05157			
Guanosine	C10H13N5O5	284.099	3	POS	FbF	MM	1	0.888134	0.128557	0.172908	0.037111	0.049914			
Hexose phosphate	C6H13O9P	259.0224	5.0-6.0	NEG	FbF	standard	1	0.758418	0.122452	0.096625	0.035349	0.027893	< 0.01	< 0.01	*
Histidine	C6H9N3O2	156.0768	12	POS	FbF	standard	1	1.487425	0.131177	0.120625	0.037867	0.034821			*
Homovanillin	C9H10O3	165.0557	1	NEG	FbF	MM	1	0.72693	0.121803	0.11861	0.035162	0.03424	< 0.01	< 0.01	*
**Homovanillinate**	**C9H10O4**	**181.0506**	**1.3**	**NEG**	**FbF**	**MM**	**1**	**0.540684**	**0.210198**	**0.169247**	**0.060679**	**0.048858**	**< 0.01**	**< 0.01**	*
Hypoxanthine	C5H4N4O	137.0458	2.5	POS	FbF	MM	1	1.76624	0.250158	0.707728	0.072214	0.204304	< 0.01	< 0.01	*
Lactaldehyde	C3H6O2	73.0295	1.3	NEG	FbF	MM	1	0.582963	0.240733	0.203323	0.069494	0.058694	< 0.01	< 0.01	*
Lactate	C3H6O3	89.02442	2.5	NEG	FbF	standard	1	1.578119	0.112186	0.217007	0.032385	0.062644			*
Leucine	C6H13NO2	132.1019	6	POS	FbF	standard	1	1.31354	0.130724	0.233272	0.037737	0.06734			*
Lysine	C6H14N2O2	147.1128	12	POS	FbF	standard	1	1.114403	0.114156	0.163702	0.032954	0.047257			
Malate	C4H6O5	133.0143	1.3	NEG	FbF	standard	1	1.129939	0.174579	0.188403	0.050396	0.054387			
Methionine	C5H11NO2S	150.0583	5.6	POS	FbF	standard	1	1.33364	0.124527	0.192535	0.035948	0.05558			*
Methionine oxide	C5H11NO3S	166.0532	9.4	POS	FbF	MM	1	1.256043	0.166721	0.12291	0.048128	0.035481			*
Methylglyoxal	C3H4O2	71.01385	1.3	NEG	FbF	MM	1	1.26533	0.195088	0.293693	0.056317	0.084782			
Methylthioadenosine	C11H15N5O3S	298.0968	4.5	POS	FbF	MM	1	2.278392	0.348528	0.983437	0.100611	0.283894	< 0.01	< 0.01	*
**Methymalate**	**C5H8O5**	**147.0299**	**1.3**	**NEG**	**FbF**	**MM**	**1**	**0.609841**	**0.243813**	**0.147464**	**0.070383**	**0.042569**	**< 0.01**	**< 0.01**	*
Ornithine	C5H12N2O2	133.0972	12	POS	FbF	standard	1	1.182166	0.15089	0.240554	0.043558	0.069442			
Oxoisocapriopoic acid	C6H10O3	129.0557	6	NEG	FbF	MM	1	1.113634	0.183726	0.333054	0.053037	0.096144			
Oxoproline	C5H7NO3	130.0499	1.5	POS	FbF	MM	1	1.473129	0.142955	0.199272	0.041268	0.057525			*
**Pentose phosphate**	**C5H11O8P**	**229.0119**	**4.3**	**NEG**	**FbF**	**MM**	**1**	**0.357521**	**0.128348**	**0.096916**	**0.037051**	**0.027977**	**< 0.01**	**< 0.01**	*
Phenylalanine	C9H11NO2	166.0863	5.5	POS	FbF	standard	1	1.441768	0.180673	0.260089	0.052156	0.075081			*
Phenylpyruvate	C9H8O3	163.0401	1.2	NEG	FbF	MM	1	0.576334	0.273181	0.174503	0.07886	0.050375	< 0.01	< 0.01	*
Proline	C5H9NO2	116.0706	8	POS	FbF	standard	1	1.686011	0.130677	0.37466	0.037723	0.108155	< 0.05		*
Propanoyl phosphate	C3H7O5P	152.9958	2.7	NEG	FbF	MM	1	0.591055	0.065748	0.048667	0.01898	0.014049	< 0.01	< 0.01	*
Propynoate	C3H2O2	68.9982	0.5	NEG	FbF	MM	1	1.190171	0.163327	0.213983	0.047148	0.061771			
Pyruvate	C3H4O3	87.00877	1.5	NEG	FbF	standard	1	1.564914	0.081177	0.1472	0.023434	0.042493			*
Serine	C3H7NO3	106.0499	7	POS	FbF	MM	1	1.742834	0.401396	0.381716	0.115873	0.110192	< 0.01	< 0.05	*
Succinate semialdehyde	C4H6O3	101.0244	1.3	NEG	FbF	MM	1	1.337701	0.258156	0.366028	0.074523	0.105663			*
Succinic acid	C4H6O4	117.0193	1	NEG	FbF	standard	1	0.567367	0.238726	0.201963	0.068914	0.058302	< 0.01	< 0.01	*
Succinylarginine	C10H18N4O5	275.135	9	POS	FbF	MM	1	1.242741	0.153697	0.262338	0.044369	0.075731			
Tartronate semialdehyde	C3H4O4	103.0037	1.2	NEG	FbF	MM	1	1.087935	0.094294	0.092593	0.02722	0.026729			
Threonine	C4H9NO3	120.0655	6.7	POS	FbF	MM	1	1.606955	0.138443	0.180327	0.039965	0.052056			*
Tryptophan	C11H12N2O2	205.0972	5	POS	FbF	standard	1	1.233881	0.100329	0.069502	0.028962	0.020064			*
Tyramine	C8H11NO	138.0913	5.7	POS	FbF	MM	1	1.554222	0.114478	0.1334	0.033047	0.038509			*
Tyrosine	C9H11NO3	182.0812	5.5	POS	FbF	standard	1	1.228824	0.16461	0.290935	0.047519	0.083986			
Valine	C5H11NO2	118.0863	6	POS	FbF	standard	1	1.245883	0.141437	0.216639	0.040829	0.062538			*

[a] Retention Time (RT)

[b] Positive Mode (POS) versus Negative Mode (NEG)

[c] Molecular Feature Extraction (MFE) extracts chromatographic peaks by molecular features versus Find by Formula (FBF) which extracts peaks by chemical formula.

[d] denotes metabolites confirmed by chemical standard (stnd). All other identifications are provisional identifications made by matching against a database of accurate mass-retention time pairs (mass-matching, MM).

[e] Univariate statistical analysis of changes (> 0.25-fold) in mean intracellular abundance by heirarchical modeling, adjusted by Benjamini-Hochberg procedure.

[f] Bivariate analysis of changes in intracellular abundance and variance by heirarchical modeling, adjusted by Benjamini-Hochberg procedure

Significance Analysis of Microarrays (SAM) analysis of changes in intracellular abundance (> 0.25-fold, FDR < 1%), significant metabolites denoted by an asterix (*)

Bold font is used to indicate those metabolites whose abundance was altered in a similar fashion in the VISA isolate from both series (SG-R and JH2) and subsequently reversed in the revertant, SG-rev, as shown in Figure 4.
